# Ultrasound-enhanced Pt-coordinated polymer immunopotentiators and heterogenic fusion membrane-based multifunctional tumor vaccine nanoplatforms for melanoma treatment

**DOI:** 10.1038/s41392-025-02355-z

**Published:** 2025-08-25

**Authors:** Ruiqian Guo, Fangxue Du, Xi Xiang, Ziyan Feng, Jianbo Huang, Chuanxiong Nie, Lang Ma, Li Qiu

**Affiliations:** 1 Department of Medical Ultrasound, National Clinical Research Center for Geriatrics, West China Hospital, Sichuan University, Chengdu, China; 2 Department of Chemistry and Biochemistry, Freie Universität Berlin, Takustraße 3, Berlin, Germany

**Keywords:** Cancer therapy, Immunotherapy, Nanobiotechnology

## Abstract

A tumor cell membrane (CM)-based biomimetic membrane tumor vaccine is an emerging prevention and treatment strategy in tumor immunotherapy. However, a single CM mostly has a weak immune-boosting effect. Here, a heterogenic fusion membrane tumor vaccine, EV–CM, was successfully constructed by fusing extracellular vesicles (EVs) from *S. aureus* and CM from B16F10 melanoma cells. Inheriting the advantages of parental components, the EV–CM combines tumor antigens with natural adjuvants that can be used for immunotherapy and can be easily synergistic with complementary therapies. In vivo vaccine tests have shown that EV–CM can activate immune antitumor responses and prevent tumorigenesis. To further enhance the immunotherapeutic and antimetastatic effects of EV–CM, Pt-porphyrin coordination polymer as an immunopotentiator (CPIP) was implanted into an EV**–**CM nanoplatform (CPIP@EV–CM), which combines localized sonodynamic/chemodynamic therapy-induced immunogenic cell death with heterogenic fusion membrane-mediated antigen-presenting functions. In vitro performance tests, cell experiments, and in vivo animal models have confirmed that the CPIP@EV–CM combined with US has better ROS production, tumor cell killing, and antimetastasis abilities. The heterogenic fusion membrane strategy and ultrasound-augmented nanoplatform present exciting prospects for designing tumor-immunogenic, self-adjuvant, and expandable vaccines, providing new ideas for exploring new melanoma immunotherapy and antimetastasis strategies, which is expected to be used as a safe and effective treatment in clinical practice.

## Introduction

Tumor immunotherapy is becoming an effective treatment method following surgery, chemotherapy, and radiotherapy.^1–5^ Personalized tumor vaccines can not only prevent the occurrence of tumors, but also be combined with other treatments to eradicate established tumors by triggering tumor-specific immune cell-mediated immunity, making them a particularly promising approach for providing long-term protection.^6,7^ By enabling targeted delivery, antigen presentation, and immune activation, tumor cell membrane (CM)-derived biomimetic nanoplatforms have surpassed traditional vaccine design protocols.^8,9^ Featuring in abundant antigens including personalized neoantigens, the CM can be recognized and uptaken by antigen-presenting cells (APCs), which further activate tumor-specific T cells and generate an anticancer immune response.^10^ Nevertheless, the low immunogenicity of mono-CM impedes the efficient immune activation of APCs, and mono-CM therefore rarely evokes an effective antitumor immune response. Hence, many efforts have been made to improve CM-based strategies.^11–14^

Over the past few years, hybrid biomimetic strategies have evolved to include fusion of homogeneous cell or membrane components to complete the codelivery of tumor antigens and immunostimulants, which is a novel approach to construct nanovaccines for tumor immunotherapy.^15,16^ For example, some natural cell membranes, including those of platelets, erythrocytes, leukocytes, and dendritic cells, have been applied as ingredients for fusion with CM for vaccine design.^17–20^ These hybrid membranes exhibit integrated functions and promote the uptake of tumor antigens via APCs.^21^ Given the complexity, high cost, and inability to isolate and culture immune or blood cells reproducibly, more facile hybrid fabrication methods have been explored to potentiate immunological functions.^22^ Bacterial extracellular vesicles (EVs), which spontaneously form membrane vesicles produced by bacteria, are considered ideal immunostimulants because they inherit many of the natural adjuvant markers from the parent bacteria in a nonreplicating form.^23,24^ Compared with Food and Drug Administration (FDA)-approved adjuvants (i.e., aluminum salt, CpG 1018, Tween 80), EVs, as natural adjuvants, can effectively activate specific immune responses.^25,26^ Moreover, they are easy to produce by culture and centrifugation procedures.^27,28^ Recently, studies have surveyed the immune function of fusion membranes prepared from CM and EVs from gram-negative bacteria (i.e., *Salmonella* and *E. coli*), and encouraging results have been obtained.^29–32^ Notably, recent studies have suggested that EVs can also be secreted from gram-positive bacteria. Unlike EVs originated primarily from the outer membrane vesicle (OMV) of gram-negative bacteria, EVs of gram-positive bacteria are mainly produced from the cytoplasmic membrane.^33^ Moreover, EVs from gram-positive *Staphylococcus aureus* (*S. aureus*) induce the production of interferon-γ (IFN-γ) and interleukins (IL-1β, IL-6), appealing as vaccine candidates. These cytokines that are closely associated with antitumor immunity.^34,35^

Although CM-based fusion membranes have great potential for initiating antitumor immunity, eradicating established tumors for monoimmunotherapy is still challenging because of the diversity, complexity, and low immunogenicity of tumors.^36^ Recently, CM-based fusion membrane strategies combined with immunotherapy and other treatment modalities have been used to more effectively ablate tumors. For example, photothermal therapy, radiotherapy, or chemotherapy can be used.^37–39^ However, these methods still face problems such as weak light penetration, high cost, and side effects, thus limiting scalability.^40,41^ Recent studies have suggested that emerging reactive oxygen species (ROS)-based therapies can not only directly kill tumor cells but also cause immunogenic cell death (ICD).^42,43^ The increasing immunogenicity of established tumor tissues induces secondary killing tumor cells by immune cells, which offers the potential to synergistically enhance antitumor immunity with fusion membrane vaccines.^44–46^ Among ROS-based therapies, sonodynamic therapy (SDT) has emerged as a potential clinical option for the treatment of a variety of solid tumors, because of its unique properties of deep tissue penetration, noninvasiveness, economy, and convenience.^47–49^ Spatiotemporally controlled SDT can accurately produce ROS in tumor tissues without damaging normal tissues.^50,51^ Moreover, chemodynamic therapy (CDT), which combats tumors through catalysis to produce ROS from overexpressed H_2_O_2_ in the tumor microenvironment (TME) without the need for external energy stimulation.^52,53^

**Fig. 1:**
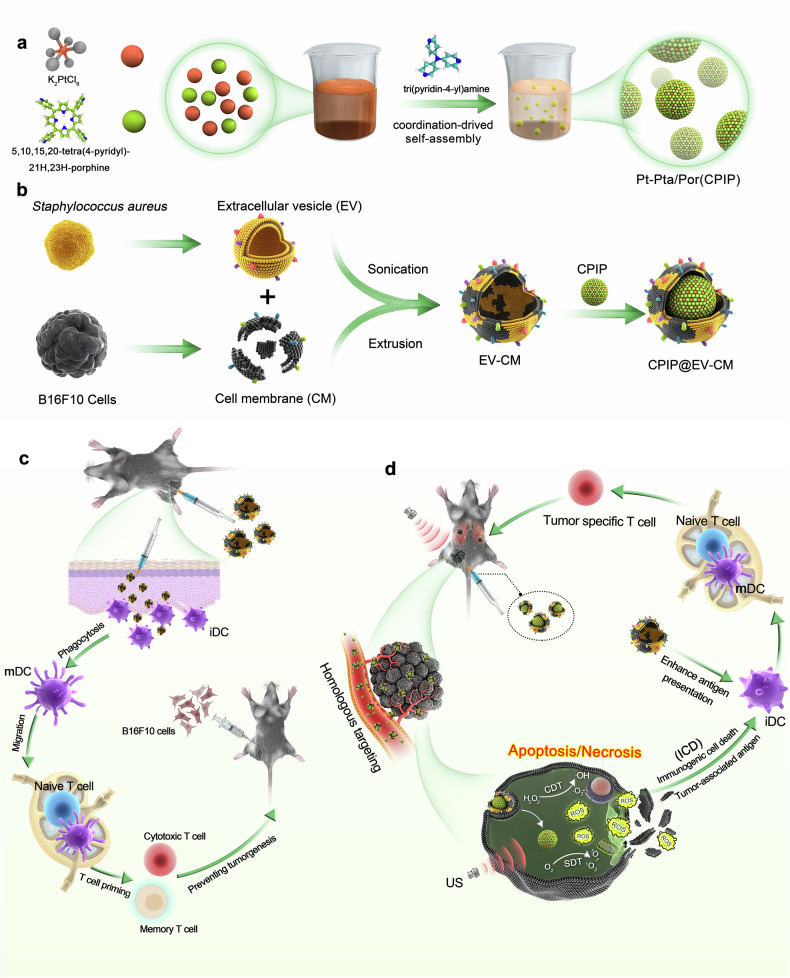
Fabrication and mechanism illustration of CPIP@EV–CM for amplifying tumor immunotherapies. **a** Assembly synthesis process of Pt-Pta/Por (CPIP). **b** Schematic illustration of the membrane derived from EV and CM fusion. The resulting hybrid membrane camouflaged with CPIP to produce CPIP@EV**–**CM. **c** The EV**–**CM hybrid membrane inhibited the occurrence of melanoma. **d** Synergistic sonodynamic/chemodynamic/immunotherapy for melanoma. Created with BioRender

In this study, by fusing EVs from *S. aureus* and CM from melanoma cells, a tumor-specific antigen vaccine with immune activation and autoadjuvant was designed. Heterogeneous fusion membrane EV–CM inherits antigenic markers from both parental components and has not only highly effective dendritic cell (DC)-based immune activation, but also cytotoxic T lymphocyte (CTL)-derived tumor-specific immunity. This integrated heterofusion membrane vaccine can provide effective prevention against tumorigenesis. Furthermore, to increase the limited tumor-eradication capacity of the heterogenic fusion membrane, the EV–CM nanovaccine is equipped with an SDT/CDT module (Fig. 1). Inspired by the excellent catalytic properties and stability of natural metal-porphyrin coordination polymers (CPs),^54^ and the fact that porphyrins are the most commonly explored biocompatible sonosensitizers,^55,56^ we designed novel Pt-porphyrin CPs for the combination of SDT and CDT. Under ultrasound (US) irradiation, Pt-porphyrin CPs can generate abundant ROS to kill tumor cells directly and act as an immunopotentiator (CPIP) to induce ICD. Verified in melanoma models, the EV–CM heterogenic nanovaccine potently suppressed tumorigenesis. Moreover, in combination with SDT/CDT, CPIP@EV–CM integrates localized ROS production and antitumor immune activation, can effectively destroy solid tumors and inhibit tumor metastasis. Our work confirms the immunotherapeutic potential of heterogeneous fusion membrane strategies and ultrasound-enhanced nanoplatforms, as well as their flexible scalability in designing tumor immunogenicity, self-adjuvant, and scalable vaccines.

## Results

### Fabrication and characterization of the EV–CM heterogenic fusion membrane

**Fig. 2:**
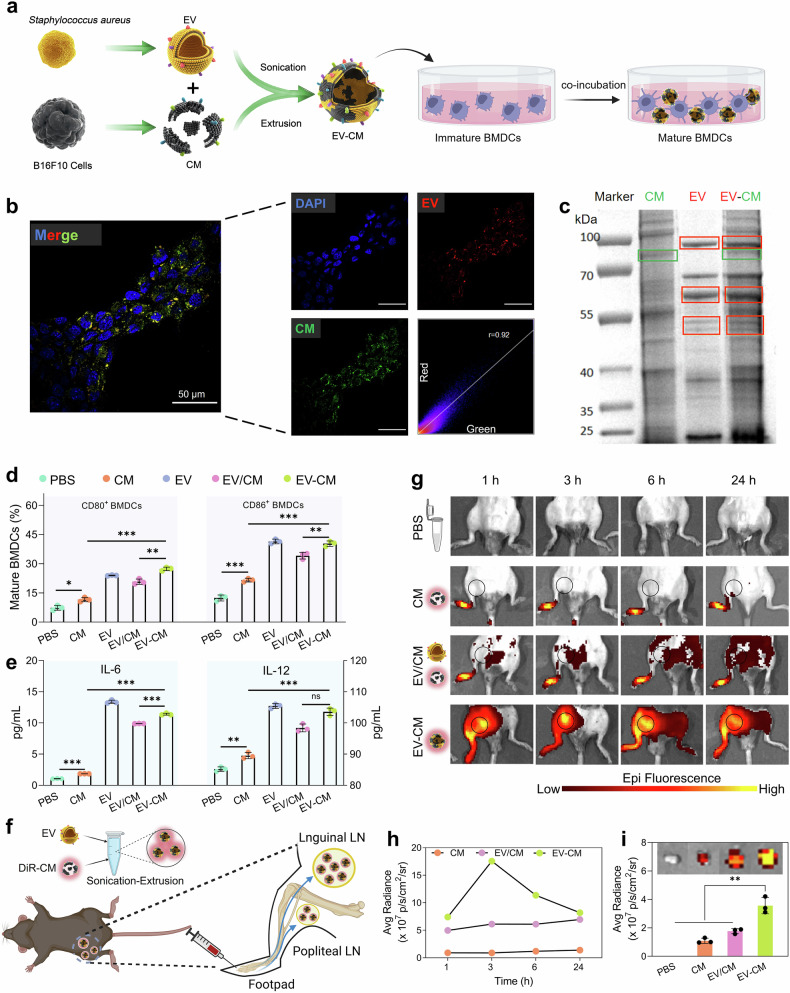
Fabrication and characterization of EV–CM and immune potential assessment. **a** Schematic illustration of the fusion membrane and immune cell activation in vitro. Created with BioRender. **b** Fluorescence colocalization images of EV–CM prepared from DiI-labeled EVs and DiO-labeled CM. **c** SDS‒PAGE protein analysis of CM, EV, and EV–CM. **d** CD80^+^ and CD86^+^ in matured BMDCs. **e** IL-6 and IL-12 from the supernatant BMDCs. **f** Schematic illustration of the migration direction of EV–CM after subcutaneous injection into the footpad. Created with BioRender. **g** In vivo fluorescence images of lymph node retention for CM, EV/CM, and EV–CM. **h** The quantitative fluorescence intensity of DiR was inspected for antigen persistence. **i** Ex vivo fluorescence images and quantitative fluorescence intensities of isolated LNs at 24 h after injection. n = 3; ns, not significant, *P < 0.05, **P < 0.01, and ***P < 0.001

We engineered an EV**–**CM heterogenic fusion membrane by fusing the B16F10 melanoma cell membrane with EVs from the gram-positive bacteria *S. aureus* (Fig. 2a). EVs were isolated from a culture of *S. aureus*, resulting in spherical membrane vesicles, and the results of protein analysis were consistent with previous literature reports (Supplementary Fig. 1).^35^ Gp100, a protein expressed specifically on the B16F10 membrane surface, was identified,^57^ indicating successful extraction of the tumor cell membrane (Supplementary Fig. 2). The protein weight ratio of CM and EVs was fused at a ratio of 2:1 by sonication and extrusion to form EV**–**CM. To verify the fusion of CM and EVs, EVs were stained with DiI (red), and the CM was stained with DiO (green). As shown in Fig. 2b, the EVs and CM exhibit distinct red and green fluorescence signals, respectively. The resulting EV**–**CM heterofusion membrane has significant colocalization of the fluorescent signal (yellow), and fluorescence colocalization analysis revealed that the degree of fluorescence overlap was high (colocalization coefficient *r* = 0.92), indicating successful fusion. Moreover, a Förster resonance energy transfer (FRET) study was used to demonstrate the fusion of two cell membranes. The EVs are labeled simultaneously with DiO/DiI and then fused with increasing amounts of CM. Fluorescence spectra revealed that the fluorescence intensities of DiO and DiI changed with the fusion of CM, indicating that the CM fusion EVs attenuated the FRET of the dyes (Supplementary Fig. 3). Furthermore, SDS‒PAGE protein analysis revealed that EV**–**CM heterogenic fusion membranes inherit primary cell membrane proteins of EVs and CM, resulting in the heterogenic fusion membrane retains the functions of EVs and CM (Fig. 2c). Notably, TEM and NTA (nanoparticle tracking analysis) indicate that EV**–**CM has good stability and that there are no significant changes in morphology, particle size or potential within 15 days (Supplementary Fig. 4).

To evaluate the DC activation response to EV-derived membranes in vitro, we incubated bone marrow dendritic cells (BMDCs) with different membrane formulations (Fig. 2a). We measured expressive costimulatory molecules and secreted cytokines via BMDCs, which can reflect the vaccine immunization-inducing antigen-specific CTL response of DCs. After incubation for 12 h, the expression of CD80 or CD86 in the CM group was only modestly upregulated. In contrast, EV-containing formulations, including EV, EV/CM (physically mixing the EV and CM), and EV**–**CM, significantly upregulated the expression of CD80 or CD86 (Fig. 2d, Supplementary Fig. 5), indicating the potent adjuvant activity of EV. Pathogen-associated molecular patterns (PAMPs) exist on the surface of EVs from *S. aureus*, and they can directly interact with pattern recognition receptors on BMDCs to stimulate DC maturation.^35^ Proinflammatory cytokines, along with antigens and costimulatory signals, constitute the third signal required for DCs to elicit a T cell response.^58^ BMDCs secreted similar levels of the proinflammatory cytokines IL-6 and IL-12, further confirming the ability of EVs to promote DC maturation (Fig. 2e). Moreover, EV**–**CM is significantly more effective than EV/CM in stimulating DC maturation. Mature BMDCs are considered to have enhanced tumor antigen presentation ability and subsequently initiate antitumor immune effects. Previous studies have suggested that integrating tumor autoantigens and immune adjuvants onto nanoparticles can effectively improve the immune activation response compared to a mixture of individual components.^59^

Given that the accumulation of the vaccine into the draining lymph nodes (LNs) and uptake by APCs is a prerequisite for immune activation, we analyzed the in vivo fluorescence intensities of CM antigens by subcutaneous injection of different membrane formulations (CM previously labeled with DiR) in the right footpad of C57BL/6 mice. (Fig. 2f). In vivo migration of the membrane formulations was investigated by fluorescence imaging within 24 h postinjection. The fluorescence intensities of the draining lymph node area (popliteal and groin) in the EV–CM or EV/CM group were consistently much greater than that in the CM group (Fig. 2g), which was confirmed by quantitative analysis of the fluorescent intensities of the inguinal LNs (Fig. 2h). These results suggest that the presence of EVs could promote CM antigen delivery. The fluorescence intensity in the EV–CM group was highest at any point in time, and more significant contralateral drainage was shown in the EV–CM group than the EV/CM group at 6 h after injection, which further demonstrated that the heterogenic fusion membrane had greater antigen delivery efficiency than did the physical mixture. Ex vivo fluorescence imaging also revealed that, compared with the other groups, the EV–CM group presented greater CM accumulation fluorescence intensity in the inguinal LNs at 24 h postinjection (Fig. 2i).

### In vivo prophylactic effects of EV–CM on melanoma

**Fig. 3:**
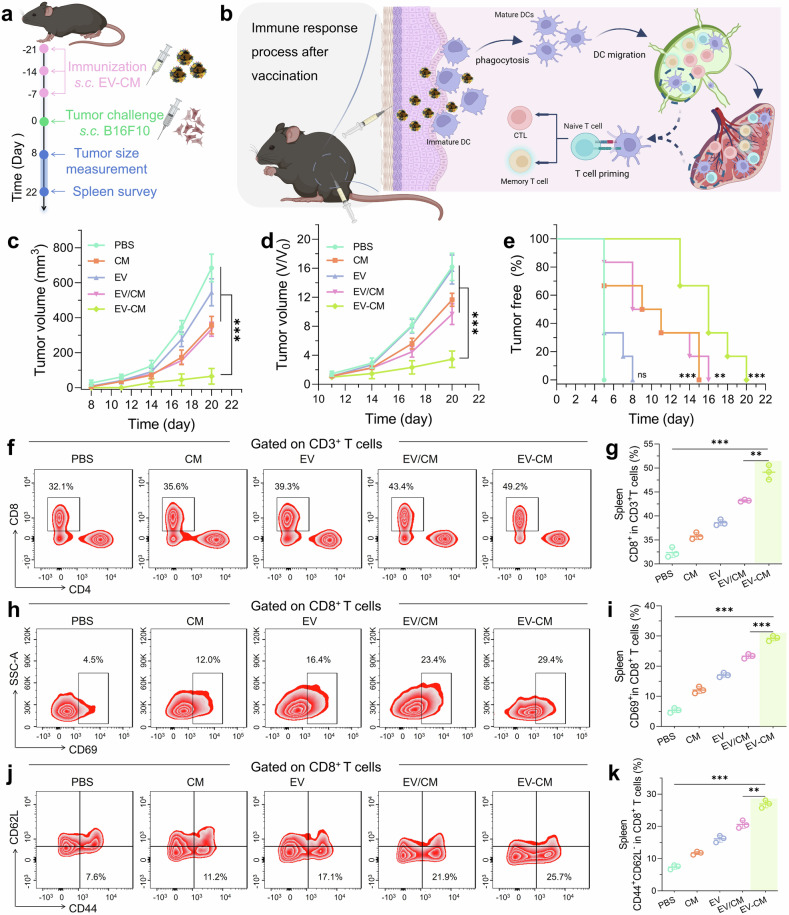
Evaluation of the preventive effect. **a** Protocol for the tumor occurrence assay. **b** Schematic illustration of antitumor immune response after subcutaneous injection of EV**–**CM. Created with BioRender. **c** Tumor growth curves after pretreatment with nanovaccines (n = 3). **d** Relative tumor volume with different nanovaccines (*n* = 3). **e** Percentage of tumor-free mice. **f** FCM plots of CD8 expression (gated with CD3) in splenic lymphocytes after immunization and **g** related quantification (*n* = 3). **h** FCM plots of CD8^+^ CD69^+^ T cells (gated with CD3^+^ CD8^+^ T cells, *n* = 3) and **i** the corresponding quantitative analysis. **j** FCM plots of EMT cells (CD3^+^CD8^+^CD44^+^CD62L^−^) in splenic lymphocytes and (**k**) the corresponding quantitative analysis (*n* = 3). ns, not significant, **P < 0.01, and ***P < 0.001. All samples used for FCM analysis were obtained at the 22nd day after injecting B16F10 cells

We conducted a tumor prophylactic trial using a melanoma model to confirm the role of EV**–**CM in initiating an antitumor immune effect and preventing tumorigenesis in vivo. As shown in Fig. 3a, the mice were subcutaneously vaccinated with different membrane formulations (CM, EV, EV/CM, and EV–CM) and then injected with B16F10 cells. Effective presentation of antigens to T cells in LNs and spleen via DCs is expected to initiate a highly effective antitumor immune effect (Fig. 3b). CM or EV/CM formulations treated groups slightly inhibited tumor growth. The EV group had few effects because of the deficiency of tumor antigen. In contrast, the EV–CM vaccine strongly suppressed tumorigenesis with a suppression rate of 78.69% (Fig. 3c, d). Furthermore, vaccination with EV–CM prolonged the tumor-free survival time (19 d postinjection) compared with that of the EV (7 d), CM (14 d), and EV/CM (15 d) groups (Fig. 3e). In the melanoma model, these formulations did not cause mortality of mice except for negligible body weight changes, suggesting good biosafety (Supplementary Fig. 6).

To survey the mechanism of prevention, we investigated a subset of T cells via flow cytometry (FCM) analysis. On the 22nd day, the collected splenocytes from mice were used to determine the number of CD8^+^ CTLs in the CD3^+^ subset. The FCM results revealed that vaccination with CM resulted in a slight increase of CD8^+^ CTLs, which was 1.11-fold greater than the control group (32.1%). High percentages of CTLs were found in both the CM/EV (43.4%) and EV–CM (49.2%) groups (Fig. 3f, g). Furthermore, the percentage of active CD8^+^CD69^+^ T cells significantly increased in the EV–CM group (29.4%) compared to the CM (12%) and control (4.5%) groups (Fig. 3h, i). The results revealed that inoculation of EVs more strongly stimulated T cell differentiation to activate cellular immune response and inflammation compared to CM.^23,31,35^ We then tested effector memory T (EMT) cells in the treatment group.^60^ The main mechanism by which EV–CM can effectively prevent melanoma is that it significantly enhances the activation of EMT (CD3^+^ CD8^+^ CD44^+^ CD62L^−^) (Fig. 3j, k).

EVs inherit many natural adjuvant components from parent bacteria which can be easily recognized by DCs and stimulate immune maturation.^36^ The implantation of EVs into CM compensates for the low immunogenicity of mono-CM, which promotes the recognition and phagocytosis of tumor antigens on CM by DCs. The CM and EVs are labeled with DiO and DiI, respectively, and compared with those in mono-CM, DCs prefer to internalize more EVs (Supplementary Fig. 7). After the implantation of EVs into CM, the internalization of CM by DCs significantly increased, manifested as a significant increase in intracellular green fluorescence. Increased CM internalization has positive effects on DC-mediated tumor antigen presentation, thereby promoting the downstream antitumor immune memory effect.

Although the EV–CM-fused nanovaccine has an encouraging inhibitory effect on tumorigenesis, existing studies have shown limited effectiveness of such monoimmunotherapies in eradicating preexisting tumors.^36^ In our work, similar results were obtained in the subcutaneous melanoma model, i.e., with a limited percentage of inhibition of tumor growth, disruption of tumor cell morphology, and tumor cell apoptosis (Supplementary Fig. 8).

### Synthesis/characterization of CPIP@EV–CM and the effects of SDT/CDT in vitro

**Fig. 4:**
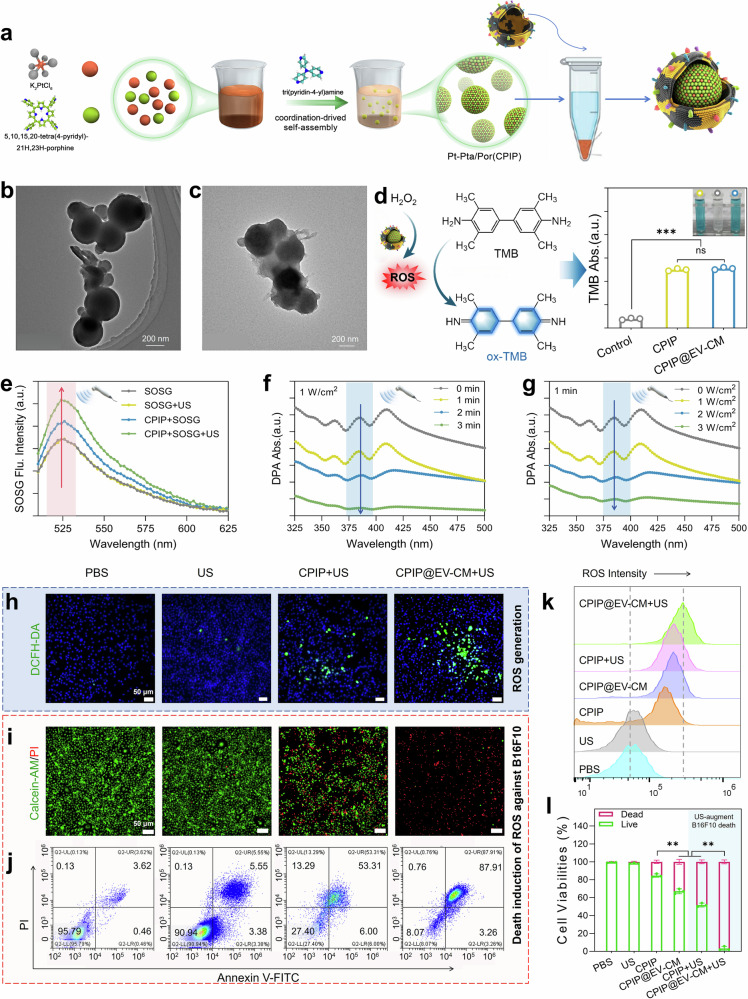
Fabrication and characterization of CPIP@EV–CM and evaluation of its direct tumor killing effect in vitro. **a** Schematic illustration of the fabrication of the CPIP@EV–CM nanovaccine. **b** TEM image of Pt-Pta/Por (CPIP). **c** TEM image of CPIP@EV–CM. **d** Schematic illustration of POD-mimicking catalytic activity and UV–vis absorbance values of the TMB assay (*n* = 3). **e** SOSG fluorescence intensity at λ = 527 nm, indicating that ^1^O_2_ was generated under US (1.0 MHz, 1 W cm^-2^). **f** Time-dependent and **g** power-dependent sono-stimulated catalytic oxidation of DPA. **h** Fluorescence images of DCFH-DA stained B16F10 cells and **k** the corresponding quantitative FCM results after incubation with the materials for 8 h. **i** Fluorescence images of B16F10 cells stained with calcein-AM/PI and **l** the corresponding quantitative analysis after incubation with the materials for 24 h (*n* = 3). **j** FCM apoptosis analysis of Annexin V-FITC/PI stained B16F10 cells after incubation with materials for 24 h. ns *P* > 0.05, **P < 0.01, and ***P < 0.001

To potentiate the tumor-elimination capacity, the EV–CM nanovaccine was equipped with an immunopotentiator (Fig. 4a). The Pt-based coordination polymer immunopotentiator Pt-Pta/Por (CPIP) was synthesized with potassium hexachloroplatinate (IV), tri(pyridin-4-yl) amine, 5, 10, 15, 20-tetra(4-pyridyl)-21H, 23H-porphine, and polyvinylpyrrolidone. The typical SEM and TEM images of CPIP exhibit an average diameter of approximately 200 nm and a particle size distribution colse to monodisperse (Fig. 4b, Supplementary Fig. 9). A uniform elemental distribution of Cl, C, N, O, and Pt in CPIP was observed via energy dispersive X-ray spectroscopy (EDS) elemental mapping images (Supplementary Fig. 10). X-ray photoelectron spectroscopy (XPS) further confirmed the detailed elements and chemical composition of CPIP. There are 1.8% (at%) of Pt content in CPIP (Supplementary Fig. 11a). Two bonding structures of Pt-N (Pt−N/pyridinic N at 399.4 eV and Pt‒pyrrolic N at 401.2 eV) in CPIP were analyzed from the N 1 *s* spectrum (Supplementary Fig. 11b). Furthermore, in the Pt 4*f* spectra of CPIP, the Pt-pyridinic N peaks at 72.3 and 75.5 eV were indexed as divalent cationic Pt. Similarly, Pt‒pyrrolic N at 74.6 and 77.8 eV was indexed as a Pt tetravalent cation (Supplementary Fig. 11c). EV–CM was subsequently coated onto the CPIP via sonication to form CPIP@EV–CM. Compared to CPIP, CPIP@EV–CM has an average diameter of approximately 252 nm with spherical core‒shell structure, of which coating membrane is ~10 nm in thickness (Fig. 4c, Supplementary Fig. 12). The EDS elemental mapping of CPIP@EV–CM revealed that P and S on the cell membrane were detected in addition to Cl, C, O, N, and Pt (Supplementary Fig. 13). Furthermore, sodium dodecyl sulfate‒polyacrylamide gel electrophoresis (SDS‒PAGE) revealed that the EV–CM was successfully encapsulated in CPIP (Supplementary Fig. 14). Within 7 d, the diameter and zeta potential remained unchanged, suggesting that the core‒shell structure of CPIP@EV‒CM was highly stable (Supplementary Fig. 15).

Next, we explored the ROS-related biocatalytic activity of CPIP@EV–CM. The POD-like catalytic conversion of H_2_O_2_ into ROS for CPIP@EV–CM was evaluated via a typical colorimetric probe, 3,3′,5,5′-tetramethylbenzidine (TMB) and the ability of CPIP@EV–CM to catalyze TMB to oxidize TMB (oxTMB) was equal to that of CPIP (Fig. 4d, Supplementary Fig. 16c). In addition, the species of ROS produced by CPIP@EV–CM with H_2_O_2_ were examined (Supplementary Fig. 16a, b). Moreover, the sonodynamic performance of the CPIP core was identified by single oxygen sensor green (SOSG). The results showed that after US irradiation, the SOSG fluorescence increased sharply (Fig. 4e, Supplementary Fig. 16d). Furthermore, as the US irradiation time or power increased, the characteristic absorption peak of DPA at approximately 378 nm decreased gradually (Fig. 4f, g), indicating that US irradiation significantly increased the production of ^1^O_2_ by CPIP@EV–CM. Our previous work demonstrated that increasing the cavitation effect could increase the generation of ROS.^61^ The US-activated CPIP@EV–CM occurs mainly through a cavitation effect that can convert the energy of ultrasonication into chemical energy for electron transfer on the metal Pt in the molecules and the porphyrin ring structure.^62^ Energy conversion can trigger a series of chemical reactions such as oxidation of the surrounding O_2_ or H_2_O molecules to generate ROS, including ^1^O_2_, ·OH, and ·O_2_^-^. These data show that the EV–CM coatings do not have a significant effect on the chemodynamic or sonodynamic performance of CPIP.

Inspired by the above excellent chemo-/sono- catalytic ROS generation properties of CPIP@EV–CM, we explored the cellular ROS production and tumor cell killing effects. We first confirmed the biosafety of CPIP@EV–CM via a CCK-8 assay and hemolysis tests (Supplementary Fig. 17). To evaluate intracellular ROS generation, a DCFH-DA probe was used to label the ROS. As expected, both CPIP and CPIP@EV–CM produced more intracellular ROS after US irradiation, as shown by the strong intracellular green fluorescence in B16F10 cells, and CPIP@EV–CM + US had the highest fluorescence intensity (Fig. 4h, Supplementary Fig. 18a), which is consistent with the results of the quantitative flow cytometric analysis of ROS generation (Fig. 4k). The direct toxicity of ROS against cancer cells was then evaluated. Both CPIP and CPIP@EV–CM induced more tumor cell death after US irradiation, demonstrating the synergistic effects of CDT and SDT in antitumor therapy (Fig. 4i, l; Supplementary Fig. 18b). Consistent with the results of live/dead staining, the results of the flow cytometric apoptosis assay revealed that cells treated with CPIP@EV–CM + US presented the highest percentage of apoptotic cells (up to approximately 90%), whereas the percentages of apoptotic cells in the CPIP, CPIP@EV–CM, and CIIP + US groups were lower (Fig. 4j, Supplementary Fig. 18c), indicating the optimal synergistic effects of CDT and SDT toxicity in the CPIP@EV–CM + US group. Notably, more cell death was induced in the CPIP@EV–CM and CPIP@EV–CM + US groups than in the CPIP and CPIP + US groups. We attribute this to the homotypic-targeting capability of CM camouflaged in EV–CM, which increases the uptake of the nanoparticle by tumor cells, followed by increased CDT and SDT effects. The bio-TEM and fluorescence labeling results for B16F10 cells supported this idea (Supplementary Fig. 19).

### Potent immune activation of CPIP@EV–CM in vitro

**Fig. 5:**
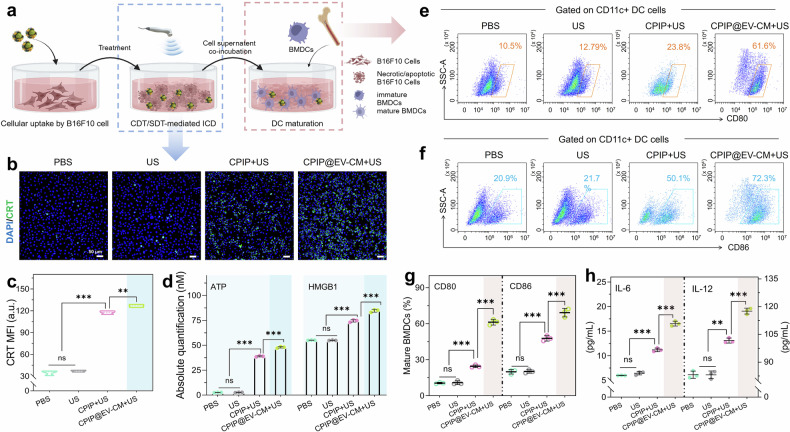
ICD effects and immune responses stimulated by CPIP@EV–CM in vitro. **a** Schematic illustration of cell uptake, SDT/CDT-induced ICD, and BMDC maturation. Created with BioRender. **b** Immunofluorescence of B16F10 cells stained with a CRT antibody and **c** the corresponding quantitative analysis after different therapies. **d** Quantification of ATP and HMGB1 release in B16F10 cells. **e** FCM analysis of the expression of CD80 and **f** CD86 of BMDCs. **g** The corresponding quantitative analysis of mature BMDCs. **h** Secretory IL-6 and IL-12 in supernatant BMDCs measured via ELISA. *n* = 3; ns, not significant, **P < 0.01, and ***P < 0.001. For panels b, c, and d of the ICD markers, the detection time point was 24 h after inoculation. For panels e, f, and g of BMDC markers, the detection time point was 12 h after the incubation of the cell supernatants

For antitumor immunotherapy, ICD induced by CDT/SDT killing of tumor cells can potentially increase the immunogenicity of tumors. To gain further insight into CDT/SDT-driven immunity, we designed a two-step coincubation using nanoparticles, B16F10 cells and BMDCs (Fig. 5a). First, ICD-related markers, including high-mobility group protein B1 (HMGB1), adenosine triphosphate (ATP), and calreticulin (CRT), were tested.^63^ ATP is a chemoattractant for antigen-presenting cells such as dendritic cells.^64^ Exposure to CRT and HMGB1 instigates phagocytosis of dying tumor cells by antigen-presenting cells and subsequent antigen presentation to T cells.^65^ When US irradiation was introduced, the green CRT fluorescence was brighter, and the CPIP@EV–CM + US group presented the brightest green fluorescence, indicating the rich expression of CRT by B16F10 cells (Fig. 5b, c; Supplementary Fig. 20a, b). Similarly, compared with those from untreated B16F10 cells, the secretion of HMGB1 and ATP from tumor cells was greatly increased, and the most significant increase was observed in the CPIP@EV–CM + US group (Fig. 5d, Supplementary Fig. 20c). These results indicate that CDT and SDT synergistically induced ICD, which was followed by APC activation, potentially boosting tumor-specific immunity.

DCs play crucial roles in innate and adaptive immunity. Immature DCs that are exposed to antigens engulf them, process them into peptides, undergo maturation, and present peptides to native T cells. Hence, we evaluated the synergistic effects of the EV–CM shell and the SDT/CDT derived from CPIP@EV–CM on DC activation by analyzing the costimulatory molecules CD80/CD86, which are considered representative markers of DC maturation. FCM revealed that DC maturity was increased to varying degrees in the treatment groups compared with that in the control group, and the highest maturation level was induced by CPIP@EV–CM + US (Fig. 5e–g, Supplementary Fig. 21a). Notably, DC maturity in the CPIP@EV–CM group was greater than that in the CPIP + US group, which contrasted with the ICD experimental results. These findings demonstrated that the effect of the EV–CM shell on BMDC activation was stronger than that of SDT/CDT-induced ICD. This further illustrates the necessity of introducing an EV–CM shell into the therapeutic system. The levels of IL-6 and IL-12, which are representative immunostimulatory cytokines produced by mature DCs, were also analyzed via ELISA, and the results were similar (Fig. 5h, Supplementary Fig. 21b). These findings confirmed that CPIP@EV–CM stimulated the maturation of immature DCs and potentially induced a subsequent immune response for immunotherapy.

### Antitumor effects of CPIP@EV–CM on established melanoma models

**Fig. 6:**
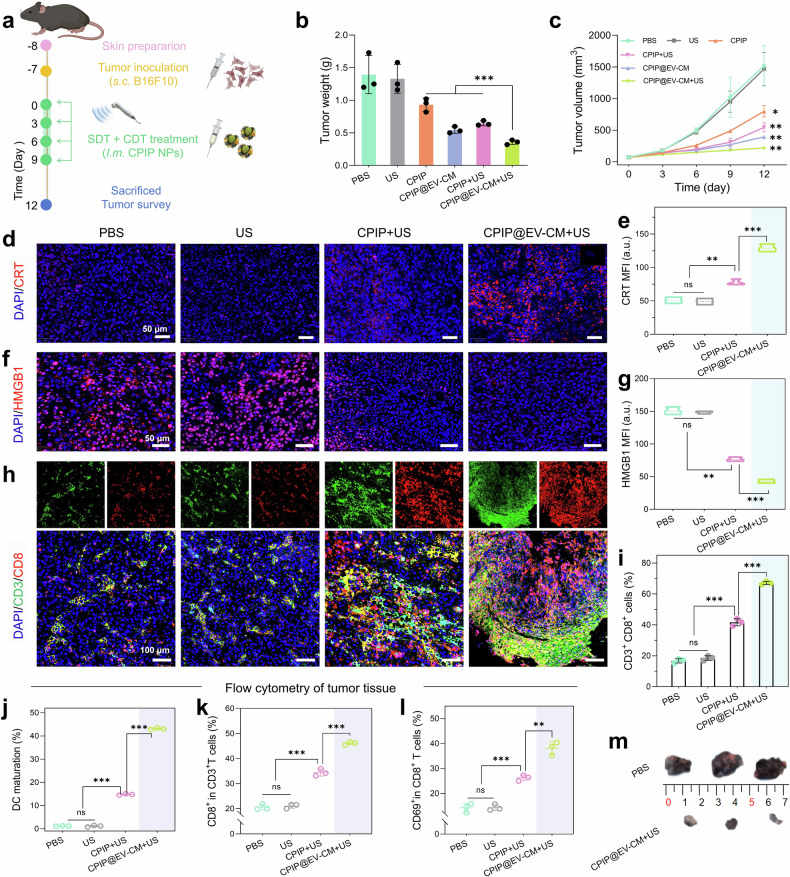
In vivo therapeutic efficacy of CPIP@EV–CM. **a** Schematic illustration of the treatment process. Created with BioRender. **b** Melanoma tissue weight at the end of treatment. **c** Growth curves of melanoma tissues. **d** Immunofluorescence images of CRT and **e** the corresponding quantitative analysis of melanoma tissues. **f** Fluorescence staining of HMGB1 in tumor sections and **g** the corresponding quantitative analysis. **h** Representative polychromatic immunofluorescence images of melanoma tissues showing DAPI (blue), CD3^+^ (green), and CD8^+^ (red) cell infiltration and **i** the CD3^+^ CD8^+^ T-cell ratio in melanoma tissues. **j** The maturation of tumor-infiltrating DCs was assessed by FCM. **k** FCM analysis showing the percentage of CD8^+^ T cells in melanoma tissues. **l** FCM image showing CD8^+^ CD69^+^ T cells in melanoma tissues. **m** Photographs of melanoma tissues. n = 3; ns, not significant, *P < 0.05, **P < 0.01, and ***P < 0.001. All samples used for pathological examination and FCM analysis were obtained on the 12th day after treatment intervention

Encouraged by the distinguished SDT/CDT and excellent activation efficacy in vitro, we investigated the antitumor performance of CPIP@EV–CM in vivo. Given the high inflammatory risk of EVs, we first assessed the concentration of C-reactive protein (CRP) and the number of white blood cells in the serum of mice after subcutaneous injection of CPIP@EV–CM. There were no appreciable differences in these indicators compared with those of the untreated mice, which confirmed the safety of CPIP@EV–CM at the selected therapeutic concentration (Supplementary Fig. 22). We subsequently generated unilateral melanoma-bearing murine models (Fig. 6a). Compared with the PBS group, the CPIP and CPIP@EV–CM groups presented a certain degree of tumor inhibition in terms of tumor volume or weight (Fig. 6b, c). Under US irradiation, the CPIP + US and CPIP@EV–CM + US groups presented superior tumor inhibition, and the CPIP@EV–CM + US group presented the highest tumor growth inhibition rate of approximately 74.4% (Supplementary Fig. 23a). Photographs of the tumor visually reflect the effect of the treatment (Fig. 6m, Supplementary Fig. 23b). At the histological level, tumor tissues in the CPIP@EV–CM + US group presented the highest ROS level among the groups, as shown by the significant red fluorescence, which was consistent with in vitro ROS assays (Supplementary Fig. 24). H&E staining of tumor tissue revealed obviously separated, sparse and fractured tumor cells in the CPIP@EV–CM + US group (Supplementary Fig. 25). Immunofluorescence staining of Ki67 and TUNEL further confirmed the effective inhibition of tumor cell proliferation by CPIP@EV–CM + US treatment (Supplementary Fig. 26 and Supplementary Fig. 27). Additionally, liver and kidney function evaluated by blood biochemistry analysis revealed no obvious abnormalities, and no noticeable organ damage was observed via H&E staining, indicating good biosafety (Supplementary Fig. 28).

Subsequently, the immune activation effect at the tumor site was assessed. Immunofluorescence staining of ICD markers, including CRT and HMGB1, was carried out in tumor tissues. Compared with PBS treatment, CPIP@EV–CM + US led to greater CRT exposure on the B16F10 cell membrane (Fig. 6d, e; Supplementary Fig. 29a). Moreover, CRT expression in the CPIP@EV–CM + US and CPIP + US groups was greater than that in the CPIP@EV–CM and CPIP groups, respectively, which suggested the synergy of SDT/CDT-induced ICD. Moreover, nuclear HMGB1 expression was also consistent with this interpretation (Fig. 6f, g, Supplementary Fig. 29b). During ICD, the gradual release of HMGB1 from the nucleus into the extracellular space resulted in a decrease in nuclear expression. Then, activated markers of DCs and T cells in tumors were evaluated by FCM. The results revealed that CPIP@EV–CM + US elicited the greatest percentage of mature DCs (CD11c^+^CD86^+^, 42.8%) (Fig. 6j, Supplementary Fig. 30a). The proportion of mature DCs in the CPIP@EV–CM + US group was 7.8, 3.0, and 1.6 times greater than that in the CPIP (5.5%), CPIP + US (14.5%), and CPIP@EV–CM (26.5%) groups, respectively. Furthermore, the percentage of CD3^+^CD8^+^ T cells obviously increased to 46.7% in tumor tissues after CPIP@EV–CM + US treatment, which was approximately 2.1-fold greater than the PBS group (21.9%) (Fig. 6k, Supplementary Fig. 30b). Additionally, many CD3^+^CD8^+^ T cells appeared in the CPIP (30.7%), CPIP + US (35.9%), and CPIP@EV–CM (38.7%) groups, indicating that SDT and CDT triggered T cell infiltration and synergized with the EV–CM shell to further enhance the immune effect. Polychromatic immunofluorescence staining images of the tumors were further confirmed by polychromatic immunofluorescence analysis, which yielded consistent results (Fig. 6h, Supplementary Fig. 31). We also tested CD69 molecule expressed on CD3^+^CD8^+^ T cells. CD69 is the earliest surface molecule expressed on activated lymphocytes and can be considered a marker of T-cell activation. There was a significant difference in the proportion of CD3^+^CD8^+^CD69^+^ T cells between the CPIP@EV–CM + US group (38.8%) and the PBS group (12.3%) (Fig. 6l, Supplementary Fig. 30c). Considering that the retention of nanomaterials in vivo might cause potential chronic toxicity, we analyzed the in vivo metabolic pathways of CPIP@EV–CM. First, the subcutaneous melanoma model was intratumorally injected with DiR-labeled CPIP@EV–CM, and images at specific time points were then acquired using an IVIS animal imaging system. CPIP@EV–CM gradually accumulated in the liver after intratumoral injection. Subsequently, the signal in the intestine gradually intensifies, and eventually gradually weakens (Supplementary Fig. 32a). Moreover, fluorescence imaging of excised major organs at different time further corroborated the in vivo imaging results. Specifically, CPIP@EV–CM aggregated in the liver and intestines and was almost completely metabolized at 24 d (Supplementary Fig. 32b). Furthermore, the relative quantitative analysis of liver signal intensity also revealed an increasing trend followed by a decreasing trend, reflecting the metabolic process of the material (Supplementary Fig. 32c). In vivo fluorescence imaging indicated that CPIP@EV–CM might be metabolized by the liver and then excreted into the intestine, where it is ultimately excreted through feces.

### Antilung metastasis effect of CPIP@EV–CM in vivo

**Fig. 7:**
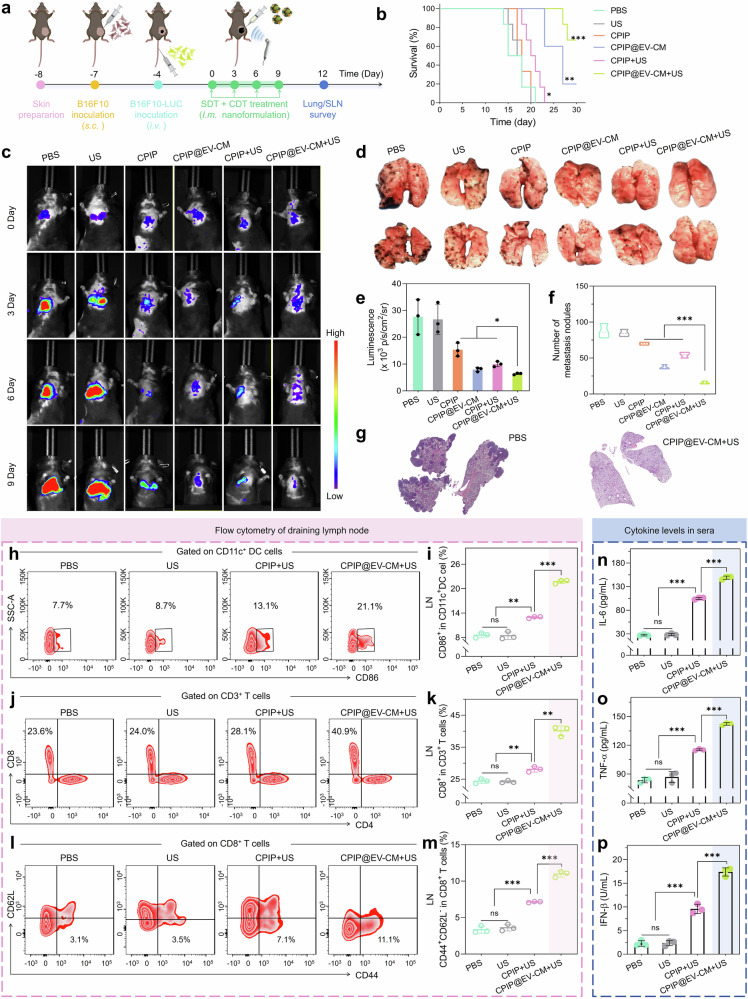
In vivo inhibiting lung metastasis efficacy of CPIP@EV–CM. **a** Schematic illustration of the lung metastasis assay. Created with BioRender. **b** Survival curves of different groups. **c** In vivo bioluminescence images of B16F10-luc for testing lung metastasis and **e** the quantitative fluorescence intensity. **d** Photographs of lung metastatic nodules and **f** the numbers of lung metastatic nodules. **g** HE-stained lung slices from the PBS and CPIP@EV–CM + US groups. **h** DCs maturation assessed via FCM (gated on CD11c) and **i** the corresponding ratio in LNs. **j** FCM analysis showing the expression of CD8 (gated on CD3) and **k** the percentage of CD8^+^ T cells in LNs. **l** FCM analysis of the proportions of EMT cells (CD3^+^CD8^+^CD44^+^CD62L^−^) and **m** the percentages in LNs. **n‒p** ELISA analysis of the concentrations of IL-6, TNF-α, and IFN-β in blood serum. *n* = 3; ns, *P* > 0.05, *P < 0.05, **P < 0.01, and ***P < 0.001. All samples used for pathological examination, FCM analysis, and ELISA were obtained on the 12th day after treatment intervention

We were concerned about whether CPIP@EV**–**CM could stop tumor metastasis. We injected B16F10-luc cells intravenously to mimic the metastasis of tumor cells (Fig. 7a). The survival rate of mice in the CPIP@EV–CM + US group increased to approximately 66%, while the survival rate of mice in the PBS, US, and CPIP groups decreased rapidly (Fig. 7b). We monitor tumor spread and growth by bioluminescence of B16F10-luc cells. Mice in the PBS group exhibit a bright bioluminescence signal and all die within 21 days (Fig. 7c). CPIP, CPIP + US, and CPIP@EV–CM had limited antitumor therapeutic effects; in contrast, CPIP@EV–CM + US had a weak bioluminescence signal and the best antitumor effect (Fig. 7e). To confirm these results, we fixed the lungs of mice with 4% paraformaldehyde and counted the number of metastatic nodules (Fig. 7d). The results revealed that the lung metastasis model of CPIP@EV–CM + US treated mice had fewer metastatic tumor nodules and achieved good antitumor effects (Fig. 7f). H&E staining of the lungs also revealed that CPIP@EV–CM + US treated mice had fewer lung metastases than those treated with PBS (Fig. 7g). Furthermore, the expression level of S100B in lung tissue, whose high expression is highly correlated with melanoma metastasis and prognosis,^66^ was visibly lower in the CPIP@EV–CM + US group than the control group (Supplementary Fig. 33). It was concluded that CPIP@EV–CM + US could effectively inhibit tumor metastasis.

To further explore the mechanism of action and synergistic treatment effect of CDT/SDT combined with EV–CM shell, LNs were collected and analyzed by FCM. The integration of EV**–**CM shells gave CPIP@EV–CM a stronger ability to promote DC maturation, making it possible to combine SDT/CDT with immunotherapy for solid tumors. Specifically, CPIP@EV–CM + US induced high proportion of DC maturation (21.1%), which was better than CPIP@EV–CM (15.6%) and CPIP + US (13.1%), indicating activation of the immune system (Fig. 7h, i; Supplementary Fig. 34a). The ratio of CD3^+^CD8^+^ T cells in the CPIP@EV–CM + US group was much greater than the control group (Fig. 7j, k; Supplementary Fig. 34b), indicating that the T cells in the LNs were significantly activated. Additionally, EMT cells were detected to further investigate the antimetastatic mechanism of CPIP@EV–CM. Notably, the proportion of CD44^+^CD62L^-^ EMT cells was markedly greater in CPIP@EV–CM + US treated group (11.1%) than PBS treated group (3.1%) (Fig. 7l, m; Supplementary Fig. 34c), indicating that CPIP@EV–CM + US could produce a strong immune memory effect and prevent tumor metastasis and recurrence in mice. In addition, before the mice were sacrificed, blood was collected and proinflammatory cytokines were measured via ELISA to evaluate the systemic inflammatory response stimulated by CPIP@EV–CM. The cytokines such as TNF-α, IL-6, and IFN-β suggested innate and adaptive immune activation (Fig. 7n-p, Supplementary Fig. 35). Collectively, these results confirmed that CPIP@EV–CM was able to improve the antitumor immunological effects of CDT/SDT treatment, indicating that CPIP@EV–CM achieved a satisfactory synergistic effect between CDT/SDT and immunotherapy for tumor suppression.

## Discussion

Personalized tumor vaccine could not only prevent the development of tumors by inducing tumor-specific immune cell mediated immunity but also could be used in conjunction with other treatments to eradicate existing tumors. CM-based biomimetic membrane tumor vaccine is a valuable choice. However, single CM mostly shows weak immune enhancement effect. To overcome this, we engineered a biomimetic nanovaccine by fusing melanoma CM with *S. aureus* EVs to enhance immune stimulation. Furthermore, recognizing that monoimmunotherapy often fails to eradicate established tumors, we integrated SDT and CDT to amplify ICD and boost systemic antitumor immunity. Our study thus addresses two critical challenges in cancer immunotherapy, which are the weak immunogenicity of CM-based vaccines and the limited efficacy of immunotherapy alone against advanced tumors.

Our findings align with and extend previous research on hybrid membrane vaccines, which have demonstrated improved efficacy by co-delivering tumor antigens and immune-stimulating adjuvants. For example, gram-negative bacterial OMVs, such as those derived from E. coli, have been widely explored for their ability to enhance DC maturation and T-cell activation. However, our work advances the field by utilizing gram-positive *S. aureus* EVs, which uniquely stimulate the production of key pro-inflammatory cytokines, including IL-1β, IL-6, and IFN-γ, all of which play pivotal roles in antitumor immunity. The fusion of EVs and CM not only improved DC uptake and activation but also enhanced lymph node trafficking, corroborating earlier reports that spatiotemporal co-delivery of antigens and adjuvants is critical for eliciting potent immune responses. Moreover, while the combination of immunotherapy with ROS-based therapies like SDT and CDT has been previously investigated, our use of CPIP as a dual-modality ROS generator represents a significant innovation. This approach enables localized tumor ablation through ROS-mediated cytotoxicity while simultaneously triggering systemic immune activation, thereby addressing the limitations of conventional monoimmunotherapy. Despite the promising prophylactic effects of the EV**–**CM nanovaccine, which achieved a remarkable 78.69% suppression of tumorigenesis in murine models, its therapeutic efficacy against pre-existing tumors remained limited when used alone. This observation is consistent with prior studies highlighting the challenges posed by tumor heterogeneity and the immunosuppressive TME. However, the integration of SDT/CDT via CPIP@EV**–**CM significantly enhanced therapeutic outcomes, achieving approximately 74.4% tumor growth inhibition and markedly reducing lung metastasis.

The underlying mechanisms of this synergistic effect were elucidated through comprehensive in vitro and in vivo experiments. First, the EV**–**CM component facilitated improved antigen presentation by promoting DC maturation and cytotoxic T lymphocyte infiltration into tumor tissues. Second, SDT/CDT-induced ICD, as evidenced by the exposure of CRT, release of HMGB1, and secretion of ATP, served as a potent immunostimulatory signal to amplify the immune response. Third, the EV**–**CM nanovaccine induced a robust memory T-cell response, which is critical for long-term immune surveillance and protection against tumor recurrence. Collectively, these findings underscore the importance of combining biomimetic vaccines with physical energy-based therapies to overcome the limitations of conventional immunotherapy. The translational potential of this study is substantial, with several key applications on the horizon. For instance, personalized vaccines could be developed by isolating CM from patient-derived tumors and fusing them with EVs to create tailored immunotherapies. Additionally, *S. aureus* EVs could serve as a broad-spectrum adjuvant platform for other cancer types or even infectious diseases, given their potent immunostimulatory properties. The use of ultrasound-responsive nanovaccines also opens the door to minimally invasive combination therapies, enabling precise tumor targeting without the need for surgical intervention. Looking ahead, future research should focus on optimizing systemic delivery strategies, such as intravenous administration, to enhance the clinical applicability of this platform. Furthermore, engineering EVs to reduce their virulence factors while retaining adjuvant activity could improve safety profiles. Finally, combining this nanovaccine system with immune checkpoint inhibitors, such as anti-PD-1/PD-L1 antibodies, may further counteract immunosuppression in the TME and enhance therapeutic outcomes.

In summary, we have developed a bioderived nanovaccine (CPIP@EV–CM) that encapsulates the SDT/CDT module by a functional hybrid membrane obtained from the melanoma cell membrane and *S. aureus* EVs. The EV–CM hybrid membrane integrates a tumor-specific antigen with a natural adjuvant, which strongly stimulates the immune system and evokes antitumor immune effects. In vivo vaccination demonstrated that the hybrid membrane protected mice from melanoma cells, demonstrating its potential as a preventive tumor vaccine. After being equipped with an SDT/CDT module (CPIP), synergistic CDT/SDT and immunotherapy with CPIP@EV–CM markebly improved the efficacy of preexisting tumor suppression, owing to both direct ROS toxicity to tumor cells and enhanced immune activation. Generally, CDT/SDT not only enhances tumor cell apoptosis and necrosis but also releases ICD-related markers to coordinate the immunostimulatory effects of EV–CM, inducing a strong antitumor immune activation in primary and metastatic tumors in mice models. Following intratumoral injection in mice, CPIP@EV–CM activated the immune system by stimulating DC maturation and T-cell activation. Furthermore, the biosafety assay results indicated that CPIP@EV–CM did not induce apparent side effects. Here, an SDT/CDT module was used for therapeutic purposes. Obviously, other support excipients with different proporties (e.g., size and morphology) and different functions (e.g., imaging, drug loading) are freely available to meet the design requirements. This work provides a versatile approach for the development of a class of cell-free vaccines. Nonetheless, to substantiate the clinical potential of the nanovaccine, incorporating a tumor rechallenge model and a more comprehensive security assessment are necessary.

## Materials and methods

### Ethics statement

All animal experiments were performed according to protocols approved by the Institutional Animal Care and Ethics Committee of West China Hospital, Sichuan University (Approval No.20221213003).

### Preparation of the B16F10 cell membrane and EVs

The B16F10 cell membrane (CM) and EVs were prepared according to previous reports.^48^ Briefly, B16F10 cells cultured to the logarithmic phase were collected and centrifuged (1500 r/min, 4 °C, 5 min) to obtain cell precipitates. Then, mixed reagents (10 mM MgCl_2_, 10 mM HCl, and 1× protease inhibitor cocktail) were used to resuspend B16F10 cells. After that, the cells were placed on ice, sonicated for 30 min, then centrifuged at 4 °C at 4000 × *g* for 10 min to collect the supernatant, followed by centrifugation at 16,000 × *g* at 4 °C for 30 min, and finally, the sediment was stored at −80 °C. Single colonies of *S. aureus* were incubated in beef extract peptone medium (BPM) (200 mL) overnight in a shaking incubator at 37 °C. Fresh BPM was added to the bacterial mixture at a ratio of 1:100 and incubated in a shaking incubator for 15 h, followed by centrifugation at 5000 ×*g* for 20 min at 4 °C to collect the supernatant. Filter the resulting mixture through a 0.45 μm vacuum filter to remove bacteria. To obtain EVs, the filtered medium was centrifuged at 150,000 × *g* for 2 h at 4 °C, resuspended in PBS, and stored at −80 °C.

### Construction of the EV–CM hybrid membrane

The membrane protein weight was determined with a bicinchoninic acid (BCA) protein kit. The fusion for CM and EVs was performed at a protein weight ratio of 2:1. First, the EVs and CM were mixed and sonicated on ice for 10 min. Then the abovementioned mixture was placed on a shaker for 40 min at 37 °C to promote membrane fusion.

Förster resonance energy transfer (FRET) assay was performed using DiO and DiI. CM was added to DiO/DiI double-stained EVs at protein weight ratios of 0:1, 1:1, 2:1, 3:1, and 4:1, followed by sonication and stirring to facilitate membrane fusion. The fluorescence recovery of the donor (DiO) at the lower emission peak was used to indicate an increasing degree of fusion.

### Synthesis of CPIP

A simple and convenient one-pot method was used to synthesize CPIP. Briefly, K_2_PtCl_6_ (19 mg, 0.039 mmol) was dissolved in deionized water (10 mL) to form solution A. 5,10,15,20-tetra(4-pyridyl)-21H,23H-porphine (1.1 mg, 0.0018 mmol), tri(pyridin-4-yl)amine (4.41 mg, 0.018 mmol), and polyvinylpyrrolidone (10 mg) were dissolved in HCl (0.01 M, 10 mL), and ultrasonicated for 30 min to form solution B. Then, solution B was fastly added to solution A and continuously stirred vigorously for 4 h. Then, the suspension was centrifuged at 11 000 r/min for 10 min, and the resulting CPIP nanoparticles were washed 3 times with deionized water and vacuum-dried at 60 °C overnight.

### Preparation of CPIP@EV–CM

CPIP suspension liquid (2.0 mL, 2 mg/mL) was mixed with the hybrid membrane mixture (2.0 mL, 2 mg/mL). The mixture was ultrasonicated for 30 min to coat the hybrid membrane onto CPIP. Afterwards, the above mixture was centrifuged at 10 000 r/min for 10 min to remove the free membrane, and then the sediment was resuspended and washed 2 times with PBS.

### Statistical analysis

Significant differences were analyzed by Student’s t test. The experiments in this work were performed at least in triplicate, and the results are presented as the means ± SDs. Significance was denoted by ns *P* > 0.05, *P < 0.05, **P < 0.01, and ***P < 0.001. All the data were analyzed with SPSS software.

## Supplementary information

Supplementary material

## Data Availability

All data needed to evaluate the conclusions in the paper are presented in the paper and/or the supplementary materials.
