# Supplementary material for: Ultrasound-enhanced Pt-coordinated polymer immunopotentiators and heterogenic fusion membrane-based multifunctional tumor vaccine nanoplatforms for melanoma treatment
Source: Signal Transduct Target Ther. 2025 Aug 25;10:278. doi: 10.1038/s41392-025-02355-z (PMC12379254; doi:10.1038/s41392-025-02355-z)
Supplement: Supplementary file 1 — Supplementary material [file 41392_2025_2355_MOESM1_ESM.docx]

Supplementary Materials for

**Ultrasound-enhanced Pt-coordinated polymer immunopotentiators and heterogenic fusion membrane-based multifunctional tumor vaccine nanoplatforms for melanoma treatment**

Ruiqian Guo^§^, Fangxue Du^§^, Xi Xiang, Ziyan Feng, Jianbo Huang, Chuanxiong Nie, Lang Ma*, & Li Qiu*

* Corresponding to: (L. Ma) malang1989@scu.edu.cn; (L. Qiu) qiulihx@scu.edu.cn

The PDF file includes:

Materials and Methods

Fig. S1 to S36

**Materials and Methods**

Materials

The Potassium hexachloroplatinate(IV) (K_2_PtCl_6_), 3,3’,5,5’-Tetramethylbenzidine, (TMB), 5,5-Dimethyl-1-pyrroline N-oxide (DMPO), and Dihydroethidium (HE) were obtained from Aladdin (Shanghai, China). 5,10,15,20-tetra(4-pyridyl)- 21H,23H-porphine (Por) and tri(pyridin-4-yl)-amine were purchased from Yanshen Technology Co., Ltd. Polyvinylpyrrolidone (PVP) and hydrochloric acid (37%) were provided by Alfa Aesar. Trypsin-EDTA was obtained from Gibco-BRL (Burlington, Canada). 3,3’-dioctadecyloxacarbocyanine perchlorate (DiO) and (1,1'-dioctadecyl-3,3,3',3'-tetramethylindocarbocyanine perchlorate) DiI were purchased from APExBIO (USA). Collagenase IV, RPMI 1640, fetal bovine serum (FBS) was purchased from Gibco (USA). 1,1-dioctadecyl-3,3,3’,3’-tetramethylindotricarbocyanine iodide (DiR), 3′-dioctadecyloxacarbocyanine perchlorate4’, 6-diamidino-2-phenylindole (DAPI), Cell counting kit-8 (CCK-8), calreticulin rabbit monoclonal antibody, FITC-labeled goat anti-rabbit IgG (H+L) , BCA protein assay kit, SDS-PAGE loading buffer, SDS-PAGE gel and phalloidin were purchased from Beyotime Biotechnology (Shanghai, China). Calcein acetoxymethyl (calcein-AM) and propidium iodide (PI) were purchased from Bestbio (China). Annexin V-FITC Apoptosis Detection Kit, hyper-sensitive chemiluminescent substrate were purchased from 4A Biotech (China). Mitochondrial Membrane Potential Detection Kit (JC-1) was purchased from Solarbio (China). CD3 rabbit monoclonal antibody were purchased from Abcam (UK). CD8 rabbit monoclonal antibody, Ki67 rabbit monoclonal antibody, HMGB1 rabbit monoclonal antibody, S100B rabbit monoclonal antibody were purchased from CST (USA). TUNEL was purchased from Promega (USA). Mouse HMGB1, TNF-α, IL-6, IL-12 and IFN-β ELISA kits were purchased from Neobioscience (China). GM-CSF protein was purchased from PeproTech (USA). Deoxyribonuclease I was purchased from Sigma-Aldrich (USA). APC anti-mouse CD11c antibody（Clone: N418）, FITC anti-mouse CD80 antibody（Clone: 16-10A1）, PE anti-mouse CD86 antibody （Clone: GL-1）, BV510 anti-mouse CD3 antibody (Clone: 145-2C11), Zombie NIR Fixable viability kit were purchased from BioLegend (USA). BUV395 anti-mouse CD45 antibody (Clone: 30-F11), BV650 anti-mouse CD4 antibody (Clone: GK1.5), PerCP-Cy5.5 anti-mouse CD8a antibody (Clone: 53-6.7) were purchased from BD (USA). Red Blood Cell Lysis Buffer and Penicillin-Streptomycin solution was purchased from Boster (China). D-Luciferin potassium was purchased from meilunbio (China). Tissue Reactive Oxygen Species (ROS) Assay kit (DHE) was purchased from BestBio (China).

*In vivo* lymph node optical imaging

To assess the migration and accumulation of CM and EV–CM in the lymph nodes, the tumor membrane was labeled with DiR to construct labeled CM and EV–CM. The labeled CM (250 μg per mouse) and EV–CM (250 μg per mouse) were administered via intraplantar injection into the right hind paw. *In vivo* live imaging was carried out with the IVIS imaging system at predetermined time intervals post injection. The mice were sacrificed at 24 h post injection, and the corresponding inguinal lymph nodes were harvested and imaged.

Prophylactic efficacy of EV–CM on melanoma

C57BL/6 mice were randomly divided into different groups and vaccinated three times at 7-day intervals via intradermal injection of 100 μL of different membrane formulations. A total of 2 × 10^5^ B16F10 cells were subcutaneously injected into the right hind flank of the mice 7 days after the last vaccination. The tumor volumes and body weights were measured every two days. The tumor volumes were calculated via a caliper according to the formula = 0.5× width^2^ × length. For immune response assays, anti-CD3 antibodies, anti-CD4 antibodies and anti-CD8a antibodies were used to test T-cell subsets in the spleen after treatment. An anti-CD69 antibody was used to test the activity of CD8^+^ cells following the standard protocol. An anti-CD44 antibody and an anti-CD62L antibody were used to test memory T cells.

Structural characterization of CPIP@EV–CM

XPS spectra were obtained via X-ray photoelectron spectroscopy (XPS, XSAM800, Kratos Analytical, UK) to determine the compositions. Transmission electron microscopy (TEM), high-resolution TEM (HRTEM), and high-angle annular dark-field scanning TEM (HAADF-STEM) were performed on a probe-corrected FEI Titan 80-300 S/TEM instrument equipped with a Gatan EELS detector. Scanning electron microscopy (SEM) was performed with a ThermoFisher Scientific Apreo SHiVoc. Sodium dodecyl sulfate‒polyacrylamide gel electrophoresis (SDS‒PAGE) was used to characterize the membrane proteins. All the samples were heated at 100 °C for 10 min after mixing with loading buffer. Afterwards, samples with equivalent protein amounts (30 µg/well) were loaded on SDS‒PAGE and run at 120 V for 4 h. The resulting PAGE gel was stained with Coomassie blue for 4 h and washed for subsequent imaging with ChemiDoc MP (Bio‒Rad, USA). The size of the NPs was measured via DLS (Zetasizer Pro Blue, Malvern Panalytical, UK).

Catalytic performance of CPIP@EV–CM

Peroxidase (POD) activity was determined via colorimetric assays in an air-saturated buffer. A total of 25 μL of catalyst (4 mg mL^−1^), 25 μL of TMB (10 mg mL^−1^), and 25 μL of H_2_O_2_ (0.1 M) were added to 2 mL of sodium acetate-acetic acid (NaOAc/HOAc) buffer [100 mM (pH 4.5)]. The catalytic oxidation of TMB (oxTMB) was studied by measuring the absorption changes of the oxidized form of TMB at *λ*_max_ = 652 nm.

Detection of ROS

HE is a specific probe that reacts with •O_2_^-^ to produce fluorescent ethidium, which is excited at 470 nm and emits at 610 nm. First, 1.5 mL of catalyst (100 μg mL^-1^) solution, 1.5 mL of H_2_O_2_ (0.1 M) and 1.5 mL of HE-ethanol solution (1 mg mL^-1^) were mixed, vortexed, and then allowed to stand for 40 min at 37 °C before fluorescence measurement (Synergy Mx, Bio Tek, USA).

TA was used as a probe, which could easily react with •OH to form a highly fluorescent product (TAOH). The catalyst (100 μg mL^-1^, 0.3 mL), TA solution (5 mM, 0.3 mL), and H_2_O_2_ (1 mM, 0.3 mL) were added to distilled water (1.8 mL), followed by mild stirring for 12 h at 37 °C in the dark. Finally, the reaction mixture was centrifuged at 10000 rpm for 2 min, and the supernatant was aspirated for fluorescence detection via fluorescence spectroscopy (Synergy Mx, Bio Tek, USA).

For ^1^O_2_ detection, 5 μL of catalyst (2 mg mL^-1^) and 30 μL of DPA-DMSO (1 mg mL^-1^) solution were added to 165 μL of PBS, followed by analysis via a UV–vis spectrophotometer. The decomposition rate of DPA by CPIP@EV–CM NPs was recorded after different US irradiation (1.0 MHz, 2.5 W cm^-2^) durations or different US irradiation (1 min) powers; the relative changes in the absorbance of DPA at 378 nm were used to quantify the decomposition rate. The US device used in this study for the *in vitro* and *in vivo* experiments is a low-intensity pulsed ultrasound with a planar transducer (Sonic-Stimu UT1021, Nu-Tek, China), which has a flexible working frequency (1 and 3 MHz) and a sound intensity range (0–3 W/cm^2^).

*In vitro* targeting of B16F10 cells

CPIP@EV–CM and CPIP were stained with DiO for 1 h. B16F10 cells were seeded and incubated for 6 h in a 24-well plate. The cells were subsequently incubated with DiO-dyed CPIP@EV–CM (100 μg/mL), DiO-dyed CPIP (100 μg/mL), or PBS. Phalloidin was added to stain the cytoplasm for 0.5 h. Then DAPI was used to stain the nuclei for 10 min. Subsequently, after washing with PBS, the cells were observed by a microscope (IX 83, Olympus, Japan). The results of the quantitative analysis of cell uptake were further analyzed via flow cytometry. After the cells were incubated with DiI-dyed NPs for 6 h, washed, and harvested, a flow cytometer was used to measure the fluorescence of DiI (Beckman Coulter, USA). Moreover, bio-TEM was used to observe the uptake of the nanoparticles in the tumor cells after incubation for 6 h.

Intracellular ROS generation

The ROS level in B16F10 cells was measured via DCFH-DA after treatment with the CPIP@EV–CM nanoagent. B16F10 cells were inoculated on 24-well plates overnight, and then CPIP@EV–CM (100 μg·mL^−1^) was added to the well and incubated for 8 h at 37 °C, followed by US irradiation (1 MHz, 1 W cm^-2^, 30% duty cycle) for 1 min. The US parameters selected were as we previously reported^1,2^. DCFH-DA was subsequently used as an ROS fluorescence probe by incubation for 30 min. Then DAPI was used to dye the nuclei of the cells. After washing with PBS, the intracellular fluorescence was monitored via an Olympus IX 83 instrument. Moreover, quantitative analysis of ROS was performed via flow cytometry (Beckman Coulter, USA).

Fluorescence imaging of live/dead cells

B16F10 cells in the logarithmic growth phase were incubated with CPIP@EV–CM (100 μg mL^-1^) for 24 h, followed by US irradiation (1 MHz, 1 W cm^-2^, 30% duty cycle) for 1 min. Then, the cells were stained with calcein-AM (AM, live cell, green color) and propidium iodide (PI, dead cell, red color) according to a standard protocol. After washing with PBS, the fluorescence was monitored via an Olympus IX 83 instrument.

Flow cytometry for cell apoptosis analysis

B16F10 cells were seeded in 12-well plates and cultured overnight. The culture medium was subsequently replaced with CPIP@EV–CM (100 μg mL^-1^) dispersed in RPMI 1640 and incubated for another 24 h, followed by US irradiation (1 MHz, 1 W cm^-2^, 30% duty cycle) for 1 min. After coincubation, the cells were collected via centrifugation. An Annexin V-FITC/PI Apoptosis Detection Kit was subsequently used to stain the cells before flow cytometry analysis. The cell populations, including live, early apoptotic, late apoptotic, and necrotic cells, were assigned to four quadrants according to their fluorescence intensity.

CRT immunofluorescent staining

To assess the expression of CRT, B16F10 cells were seeded on 24-well plates at a density of 5 × 10^4^ well^−1^ and cultured for 12 h. Then, CPIP@EV–CM (100 μg mL^-1^) was added to the cells and incubated for another 24 h, followed by US irradiation (1 MHz, 1 W cm^-2^, 30% duty cycle) for 1 min. After being washed with PBS three times, B16F10 cells were fixed with 4% paraformaldehyde and blocked with Immunol staining blocking buffer. Then, the B16F10 cells were incubated with a rabbit monoclonal calreticulin antibody at 4 °C overnight. Then, the cells were washed three times with TBST, followed by incubation with a FITC-labeled goat anti-rabbit IgG (H+L) antibody for 1 h. After being stained with DAPI, the cells were visualized via a microscope (IX 83, Olympus, Japan) and quantified via ImageJ software.

Detection of HMGB1 and ATP release

HMGB1 concentrations in the supernatants of the cells following the indicated treatments were measured via ELISA according to the manufacturer’s protocol (Neobioscience, China). The ATP concentrations in the supernatants of the cells subjected to the indicated treatments were measured via an ATP assay kit according to the manufacturer’s protocol (Beyotime, China). Luminescence and absorbance were measured by using a microplate reader (Synergy Mx, Bio Tek, USA).

Flow cytometry analysis of BMDC activation *in vitro*

BMDCs were isolated from the bone marrow of 6-week-old C57BL/6 mice according to established protocols^3^. First, femur and tibia bones were isolated from healthy C57BL/6 mice. Then, both ends of the bone were cut, and the bone marrow was flushed out by slowly injecting RPMI 1640 culture medium. The collected bone marrow was filtered through a 70 μm cell strainer to a 50 mL centrifuge tube, followed by centrifugation at 400 ×*g* for 5 min and subsequent lysis of red cells. The obtained cells were resuspended in RPMI 1640 culture medium supplemented with 20 ng/mL GM-CSF and 0.05 mM β-mercaptoethanol and then incubated at 37 °C and 5% CO_2_ and the culture medium was half-changed every two days. Afterwards, the BMDCs were harvested by collecting nonadherent and loosely adherent cells from the suspension.

To study the effects of different membrane formulations on BMDC maturation, CM (100 μg mL^-1^), EV (50 μg mL^-1^), EV–CM (100 μg mL^-1^), and EV/CM (100 μg mL^-1^) were incubated with BMDCs for 12 h. The supernatants were collected for further cytokine analysis. The cells were washed three times with PBS and then stained with anti-CD11c, anti-CD86, and anti-CD80 antibodies for 15 min at 4 °C. After being washed with cold PBS three times, the fluorescence of the cells was analyzed via flow cytometry.

To study the effect of EV–CM combined with SDT/CDT on BMDC maturation. B16F10 cells were first seeded on 24-well plates at a density of 5 × 10^4^ cells/well and then cultured overnight for cell attachment. Then the cells were incubated with CPIP@EV–CM (100 μg mL^-1^) for 24 h, followed by US irradiation (1 MHz, 1 W cm^-2^, 30% duty cycle) for 1 min. Then the cell supernatants from the different treatment groups were collected and added to the cultured BMDCs. The BMDCs were then cultured for 12 h, followed by staining with ith APC anti-mouse CD11c, FITC anti-mouse CD80 and PE anti-mouse CD86 antibodies for 30 min. After washing three times, the cells were analyzed via FCM (Beckman CytoFLEX, USA).

The antitumor immunity effect in B16F10 orthotopic tumor models

C57BL/6 mice (6 w) were purchased from GemPharmatech Co., Ltd. To establish the animal melanoma model, B16F10 cells (approximately 3×10^5^) were injected into the subcutaneous layer of the right hind flank with a fine needle to form a homogeneous dermatocollus. After the tumor volume reached approximately 100 mm^3^ (approximately 8 days), the mice were randomly divided into six groups (n = 3 per group), and tumor treatment was carried out as follows (Day 0): (1) PBS, (2) US, (3) CPIP, (4) CPIP+ US, (5) CPIP@EV–CM, and (6) CPIP@EV–CM+US. The tumor-bearing mice were intratumorally injected with PBS or different nanoagents (10 mg kg^-1^). The injection was conducted every 3 days, followed by ultrasonic irradiation. The ultrasonic conditions were as follows: intensity (2.5 W cm^-2^), frequency (1 MHz), duty cycle (30%), and time (3 min), the selection of which was previously reported^1^. The timing of US exposure was determined on the basis of *in vivo* fluorescence imaging. First, the tumor-bearing mice were intratumorally injected with DiR-labeled CPIP@EV–CM, and then an IVIS animal imaging system was used to obtain images at specific time points. As shown in Fig. S36, after intratumoral injection for 4 h, the fluorescence signal peaked, which was the appropriate time point for US intervention. Tumor volume (calculated as length × width^2^/2) and body weight were monitored via a Vernier caliper or [weighing machine](javascript:;) before each treatment. A curve of the relative tumor volume change was drawn. The TGI was calculated as follows: TGI = (tumor volume in the control group – tumor volume in the experimental group)/tumor volume in the control group × 100%. On day 12, all the mice were sacrificed, and HE staining was performed to analyze tissue damage after various treatments. DHE staining of frozen tumor sections was carried out to evaluate the ROS level in tumor tissues. In addition, Ki67 staining was carried out to verify cancer cell proliferation, and TUNEL staining was carried out to verify apoptosis. HMGB1 and CRT staining were carried out to verify immunogenic cell death. Moreover, the infiltration of CD8^+^ T cells in tumors was observed via polychromatic immunofluorescence staining. To assess tumor-infiltrating immune cells more comprehensively, the tumors in each group were immersed in 5 mL of tissue lysates containing collagenase IV and DNase I for 30 min at 37 °C and then passed through a 70 μm filter to harvest single-cell suspensions. Then, flow staining was performed according to standard protocols and flow cytometry was used for detection and analysis (BD Symphony A5, USA).

Inhibition of tumor metastasis in B16F10 cell-bearing mice

To evaluate the ability of CPIP@EV–CM to inhibit tumor metastasis, a pulmonary metastasis model was established via tail vein injection of B16F10-Luc cells (1 × 10^6^ cells/mouse) on day -4. The subsequent treatments were the same as those described above. Before each treatment, an *in vivo* imaging system (AniView100, Biolight, China) was used for bioluminescence imaging of the mice 15 min after the intraperitoneal injection of D-Luciferin potassium. On Day 12, the mice were sacrificed, and their lungs were photographed to evaluate the number of metastatic nodules on the surface (frontal and dorsal). HE staining and S100B immunohistochemical staining of the lungs were carried out to assess the extent of lung metastasis. To assess systemic antitumor immunity, the lymph nodes were isolated and ground. The single-cell suspensions were harvested and then subjected to flow staining according to standard protocols. Moreover, blood samples were collected before the mice were sacrificed, and the levels of proinflammatory cytokines, including TNF-α, IL-6, and IFN-β, were determined via ELISA.

Cell and bacterial cultures

Murine melanoma cancer B16F10 cell were purchased from American Type Culture Collection (ATCC) and cultured in the medium of RPMI 1640 containing 10% fetal bovine serum (FBS), and 1% antibiotics mixture (10,000 U penicillin and 10 mg streptomycin) at 37 ℃ under 5% CO2.

S. aureus were purchased from American Type Culture Collection (ATCC 12600) and cultured in the Beef extract Peptone Medium (BPM) medium (200 mL) in a shaking incubator at 37 °C.

*In vitro* cytotoxicity

Human Umbilical Vein Endothelial cells (HUVECs) or Bone Marrow-Derived Dendritic Cells (BMDCs) were seeded on 96-well plate (1 × 10^4^ cells/well). When achieving 80-90% confluence, the medium was replaced by a medium containing different concentrations of CPIP, EV–CM or CPIP@EV–CM and then incubated for 24 h. Cell counting Kit-8 (CCK8) assay was performed according to a standard protocol and cell viability was evaluated by measuring the absorbance of the reaction products at 450 nm using a microplate reader (Synergy Mx, Bio Tek, USA). Every experiment was conducted 3 times.

Hemolysis test

Blood cells were collected from C57BL/6 mice and incubated with different amounts of EV–CM, CPIP, and CPIP@EV–CM in tubes at 37 °C. After 60 min, the samples were centrifuged to examine the hemolysis. The blood cells in saline and the blood cells in distilled water were used as controls.

Supplementary figures


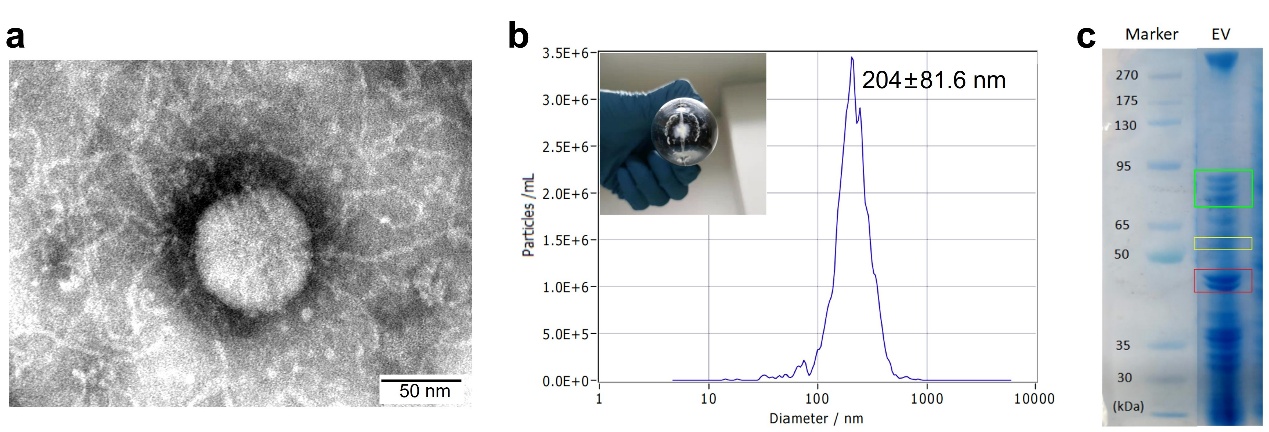


**Fig. S1 Characterization of EVs derived from *S. aureus*.**

**a** The TEM of EVs from *S. aureus.* **b** The hydrodynamic size of EVs. **c** The SDS-PAGE protein analysis of EVs.


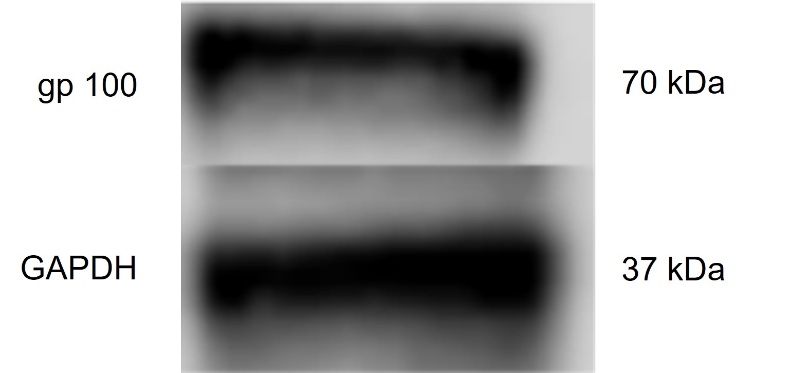


**Fig. S2 Immunoblotting analysis of the CM.**

The Gp100, an antigen expressed specifically on the B16F10 membrane, indicating the successful extraction of tumor cell membrane.


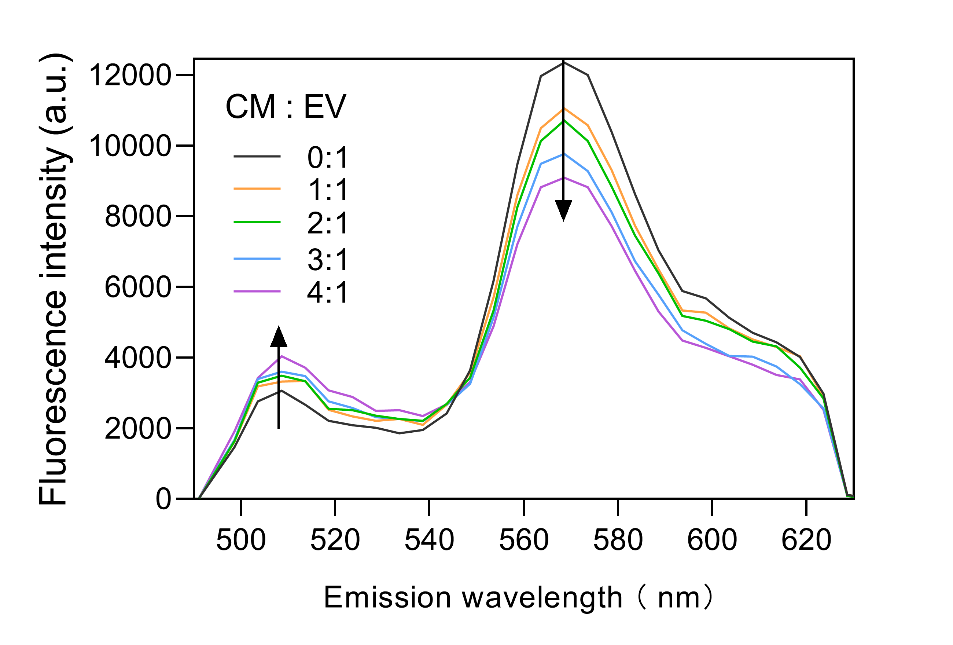


**Fig. S3 A pair of Förster resonance energy transfer (FRET) fluorescence dye DiO and DiI is chosen to conduct the fusion process of two membranes.**

The EVs is doped with two fluorescence dye and fused with increasing amounts of B16F10 cell membrane (CM).


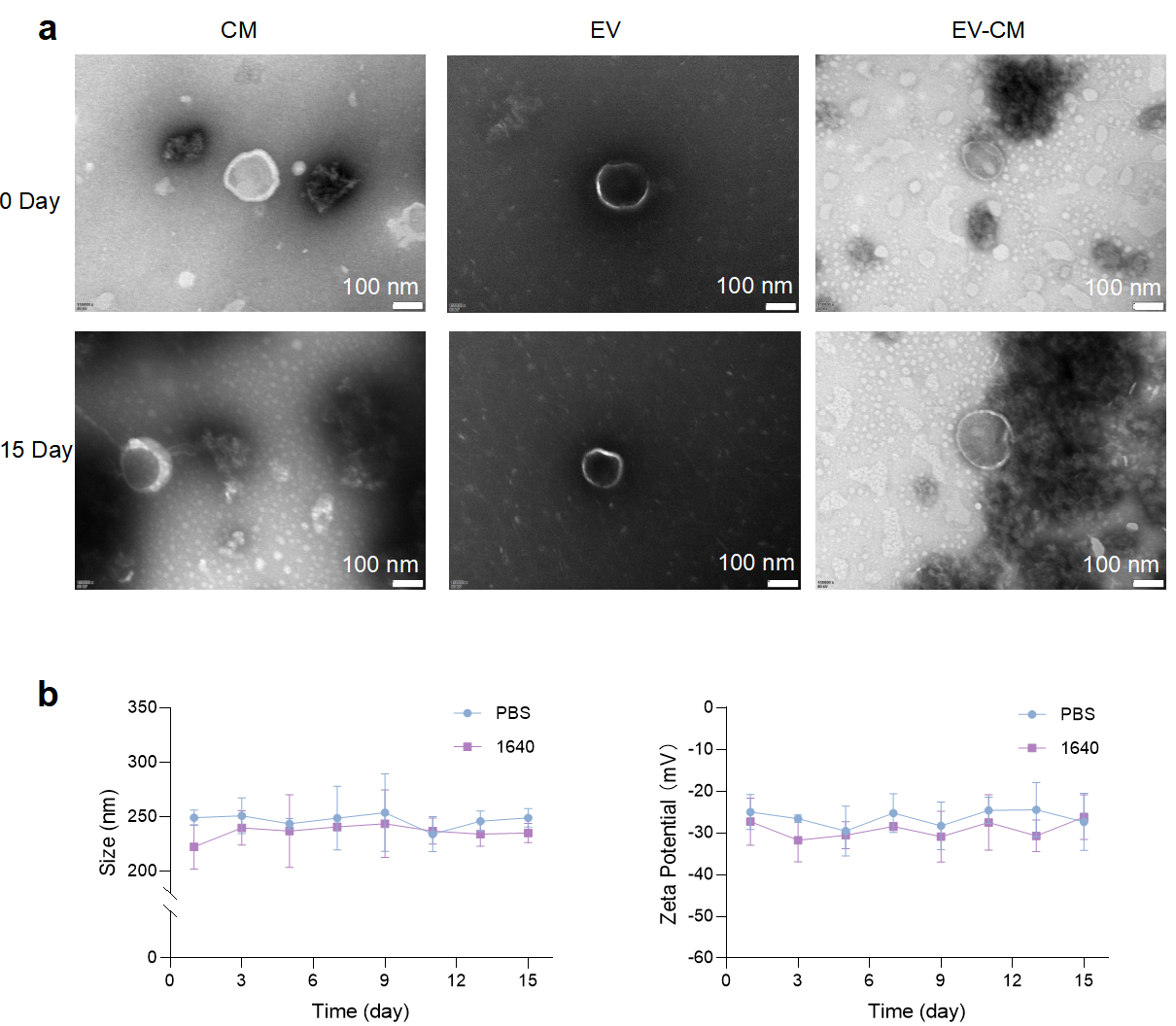


**Fig. S4 The characterizations of EV**–**CM.**

**a** The TEM of CM, EV, and EV–CM. **b** The particle size and potential of EV–CM in different solvents detected by NTA.


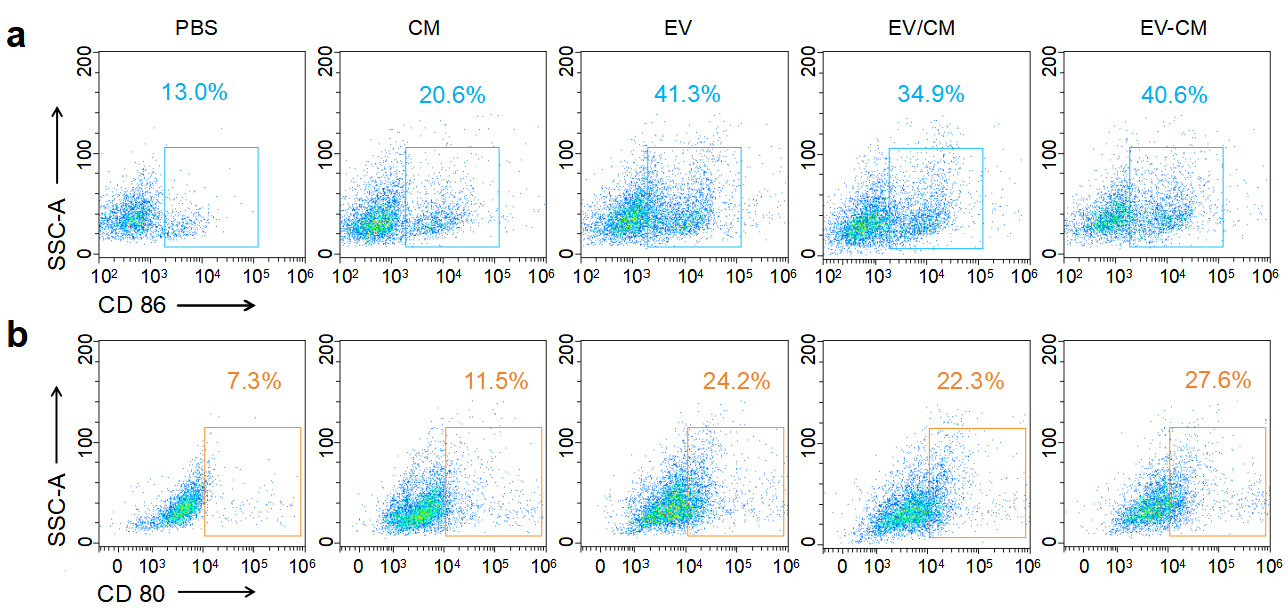


**Fig. S5 DC activation after *in vitro* incubation of BMDCs with different membrane formulations.**

**a** and **b** Flow cytometry analysis of the expressed CD80 and CD86 on BMDCs after incubated with different membrane formulations for 12 h.


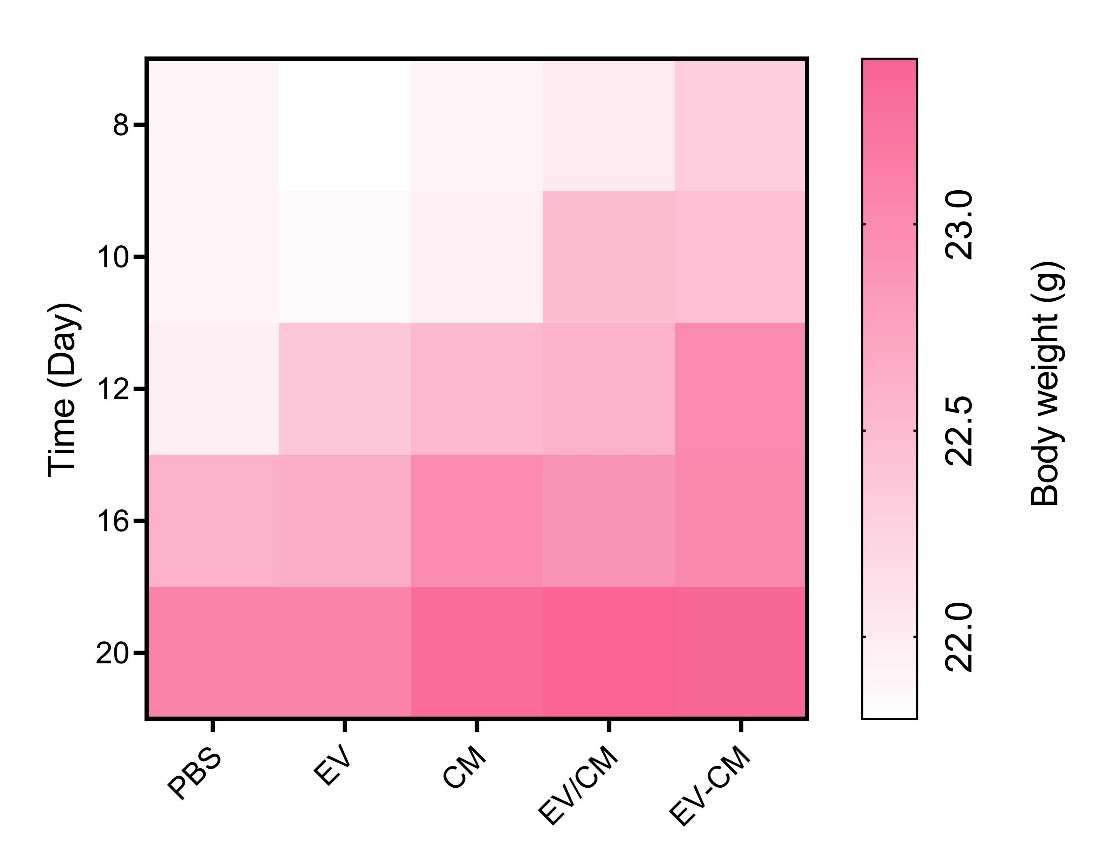


**Fig. S6 Heat map of mouse weight.**

Body weight change of mice from different groups during the whole treatment.


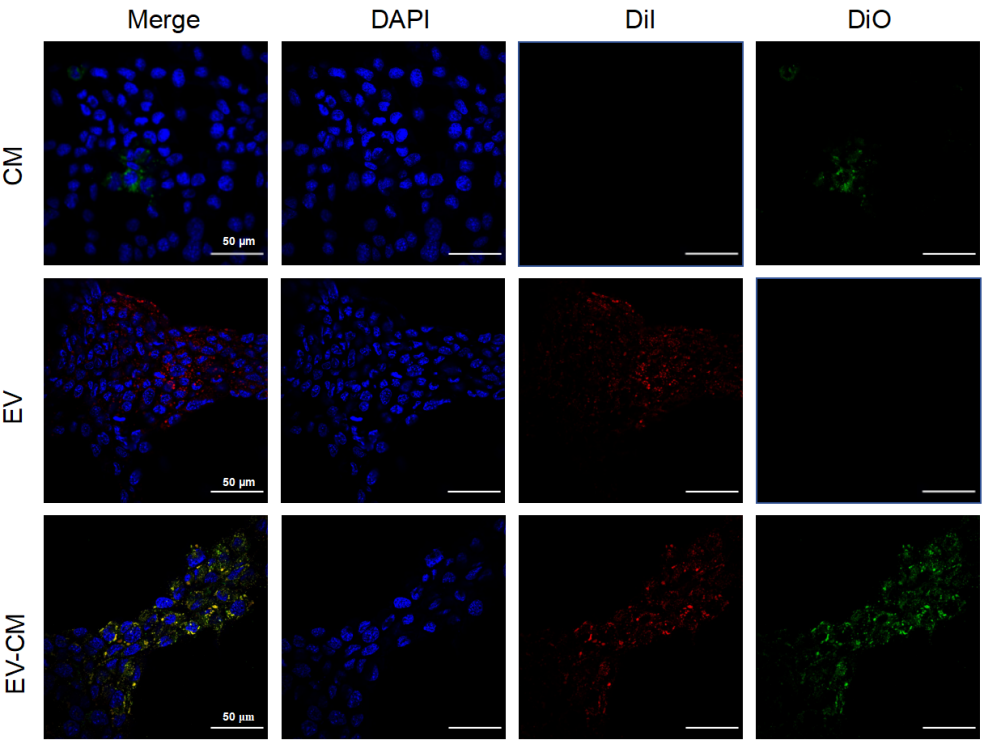


**Fig. S7** **CLSM observation of DC2.4 endocytosis.**

The CM and EV were labeled with DiO and DiI, respectively. EV–CM prepared from DiO-labeled CM and DiI-labeled EVs. This figure is related to Fig. 2b.

**
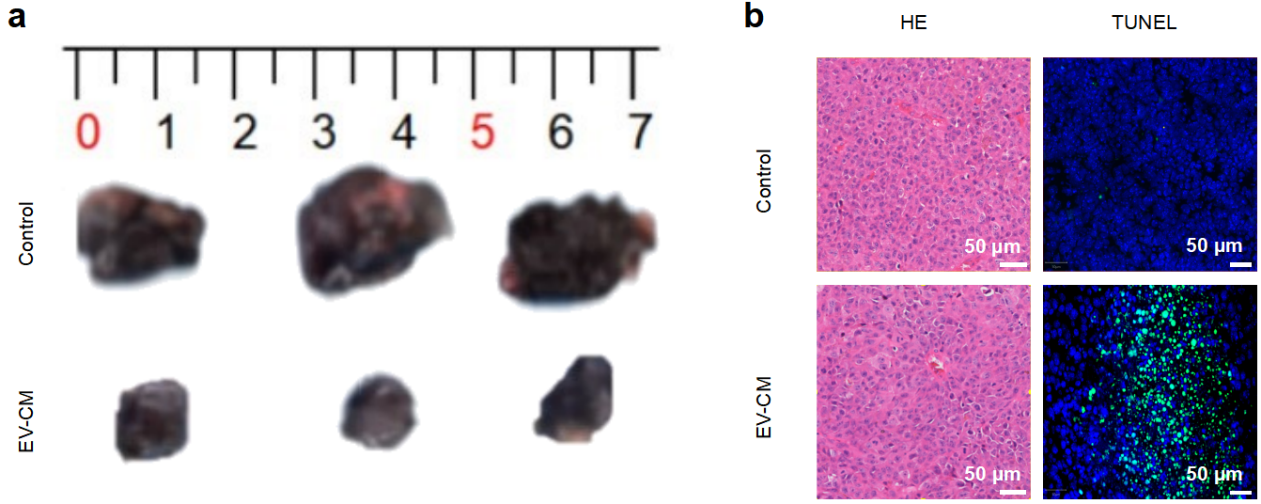
**

**Fig. S8 Limited effectiveness in** **eradicating pre-existing tumors for EV**–**CM fused nanovaccine.**

**a** Tumor photographs collected from the mice after indicated treatments in subcutaneous melanoma tumor model. This figure is related to Fig. 6m and Fig. S23b. **b** Images of HE, TUNEL staining of tumor sections from indicated treatment groups in subcutaneous melanoma tumor model. All samples used for pathological examination were obtained on the 12th day after treatment intervention. This figure is related to Fig. S25, 26.


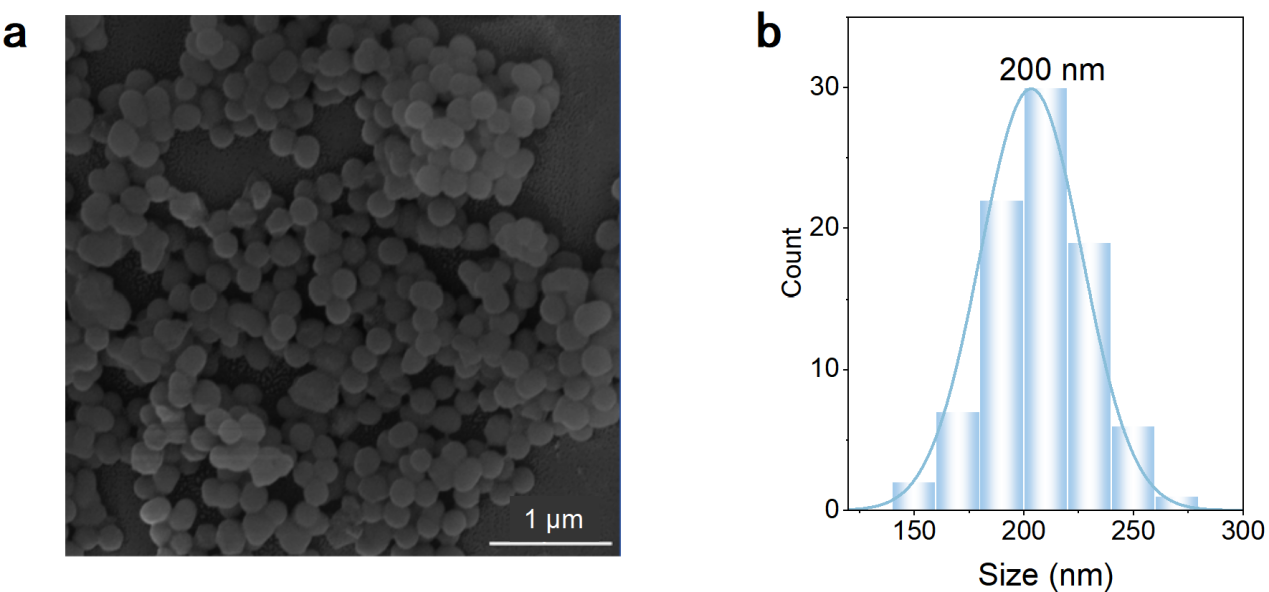


**Fig. S9 Characterization of Pt-Pta/Por (CPIP).**

**a** Scanning electron microscopy image of CPIP. **b** The corresponding average sizes of the CPIP.

**
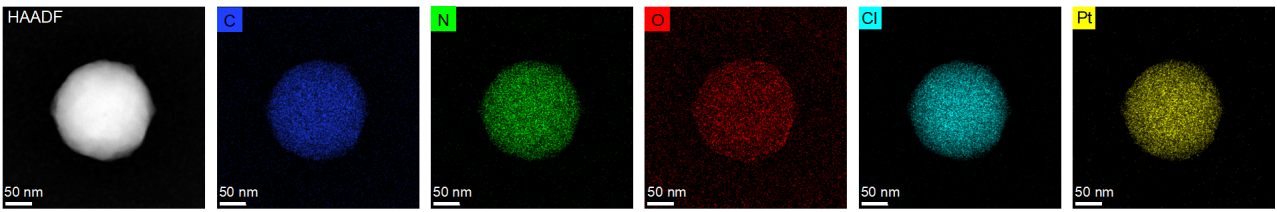
**

**Fig. S10** T**he energy dispersive X-ray spectroscopy (EDS) elemental appings of CPIP.**

Blue, elemental C; green, elemental N; red, elemental O; cyan, elemental Cl; and yellow, elemental Pt.


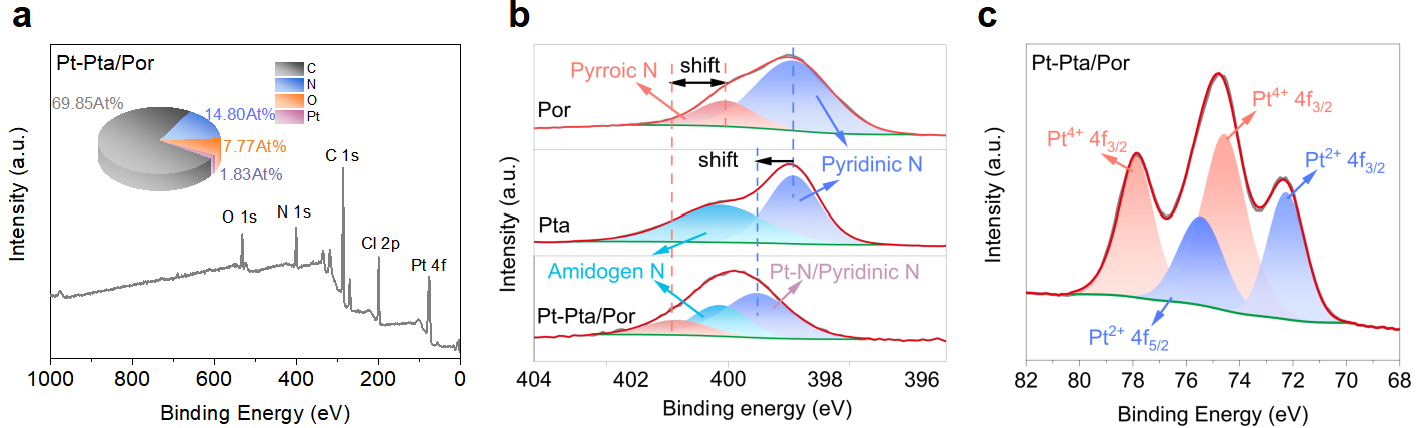


**Fig. S11 The structural characterizations of Pt-Pta/Por (CPIP).**

**a** XPS survey scanning of Pt-Pta/Por. **b** N 1*s* XPS spectra for Por, Pta, and Pt-Pta/Por. **c** Pt 4f XPS spectra for Pt-Pta/Por.


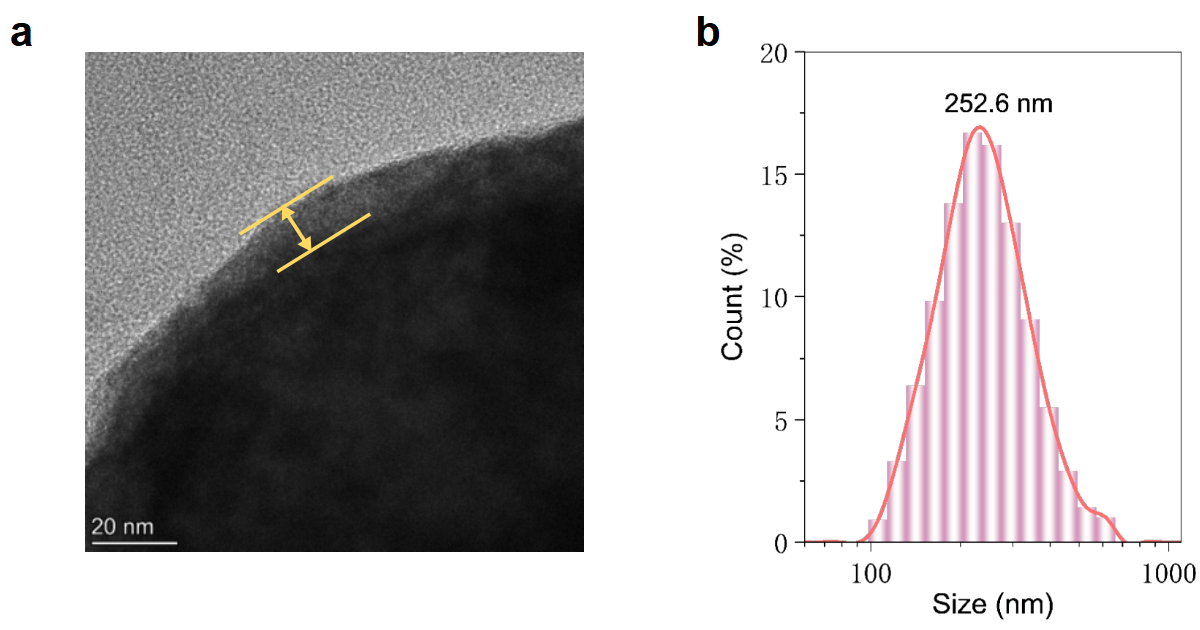


**Fig. S12 Characterization of CPIP@EV**–**CM.**

**a** TEM image of CPIP@EV–CM. Yellow lines indicate the thickness of the EV–CM membrane. **b** The corresponding average particle sizes of CPIP@EV–CM in PBS.


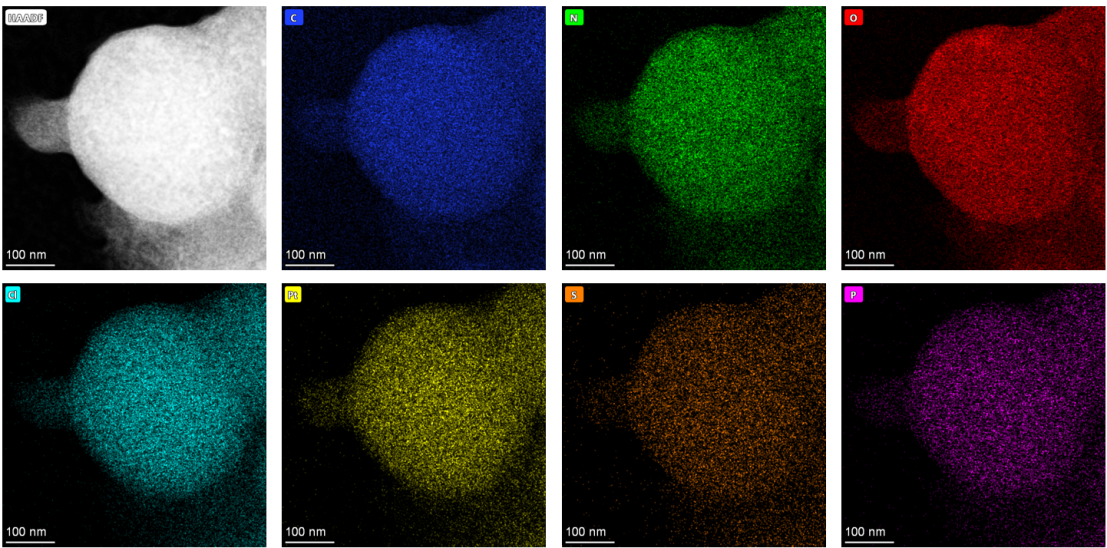


**Fig. S13** T**he energy dispersive X-ray spectroscopy (EDS) elemental appings of CPIP@EV**–**CM.**

Blue, elemental C; green, elemental N; red, elemental O; cyan, elemental Cl; yellow, elemental Pt; orange, elemental S; and purple, elemental P.


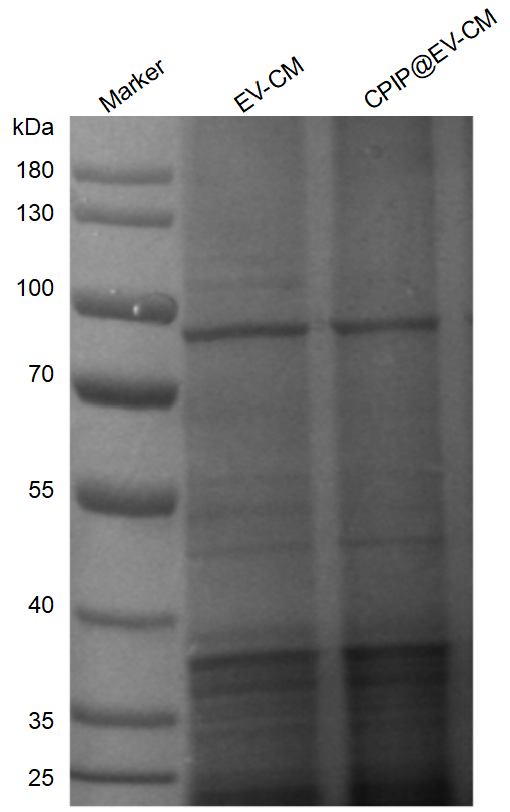


**Fig. S14 SDS-PAGE protein analysis of protein retained on the EV**–**CM and CPIP@EV**–**CM.**

The protein on CPIP@EV–CM and EV–CM membranes exhibit similar profiles, suggesting that the profiles are entirely retained in the CPIP@EV–CM.


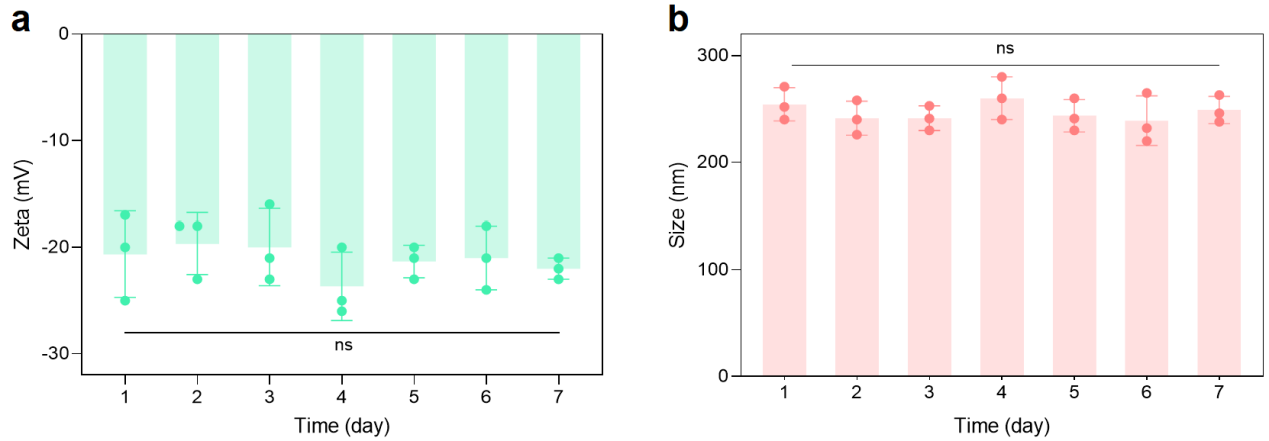


**Fig. S15 The stability of CPIP@EV**–**CM.**

**a** Mean ζ potential of CPIP@EV–CM in PBS. **b** The particle size of CPIP@EV–CM in PBS. (ns *P* > 0.05).


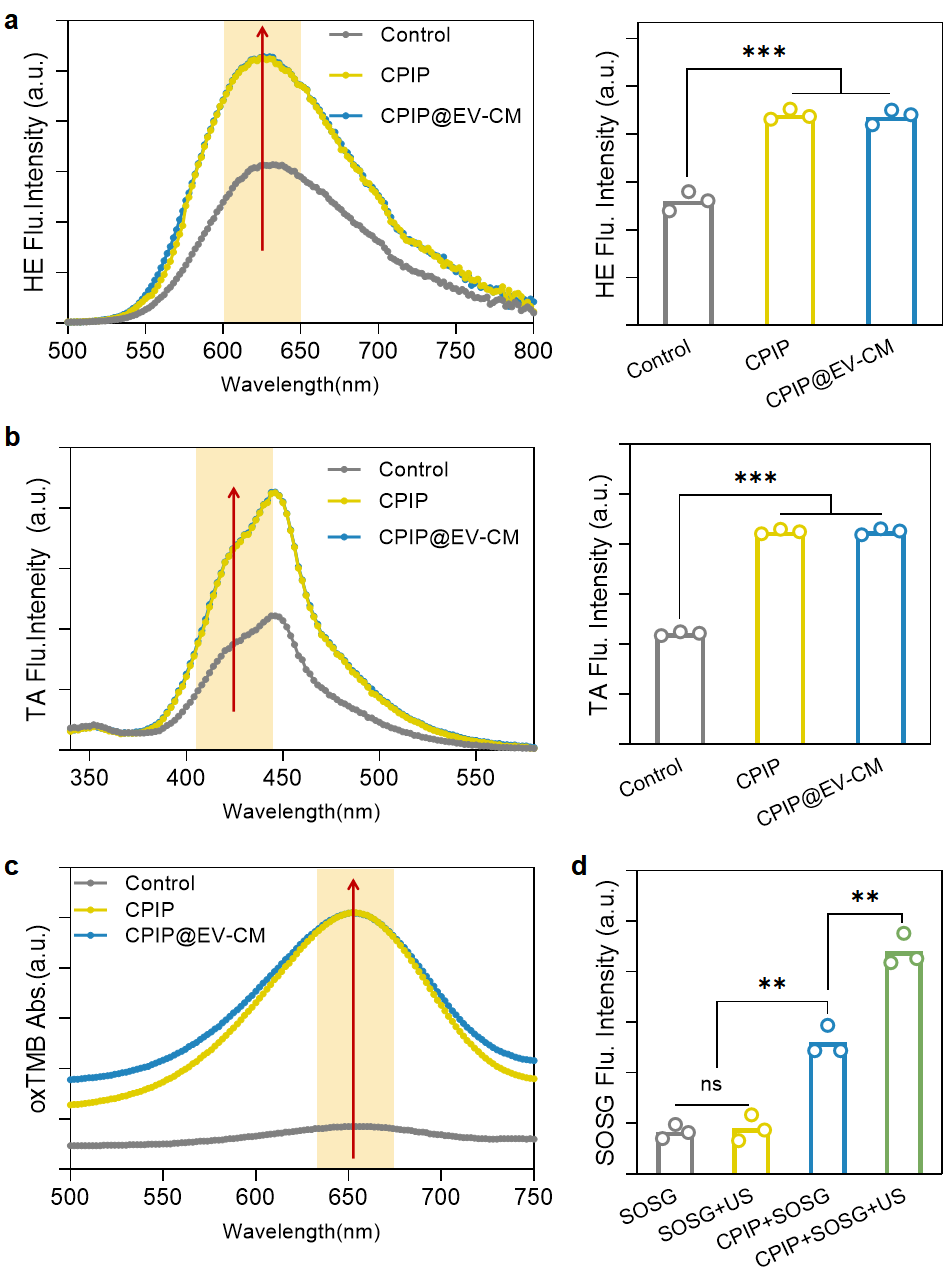


**Fig. S16** **The catalytic characterizations of CPIP@EV**–**CM.**

**a** •O_2_^-^ generation of CPIP@EV–CM with HE+H_2_O_2_ as the substrate, and the quantitative analysis of absorbance values at λ= 625 nm. **b** •OH generation of CPIP@EV–CM with TA+H_2_O_2_ as the substrate, and the quantitative analysis of absorbance values at λ= 425 nm. **c** UV-vis absorption spectra of the catalyzed oxidation of TMB (oxTMB). **d** ^1^O_2_ generation of CPIP with SOSG as the substrate, fluorescence intensity at λ= 527 nm indicating ^1^O_2_ generated under US (1.0 MHz, 1 W cm^-2^). (ns *P* > 0.05, ***P* < 0.01, ****P* < 0.001)


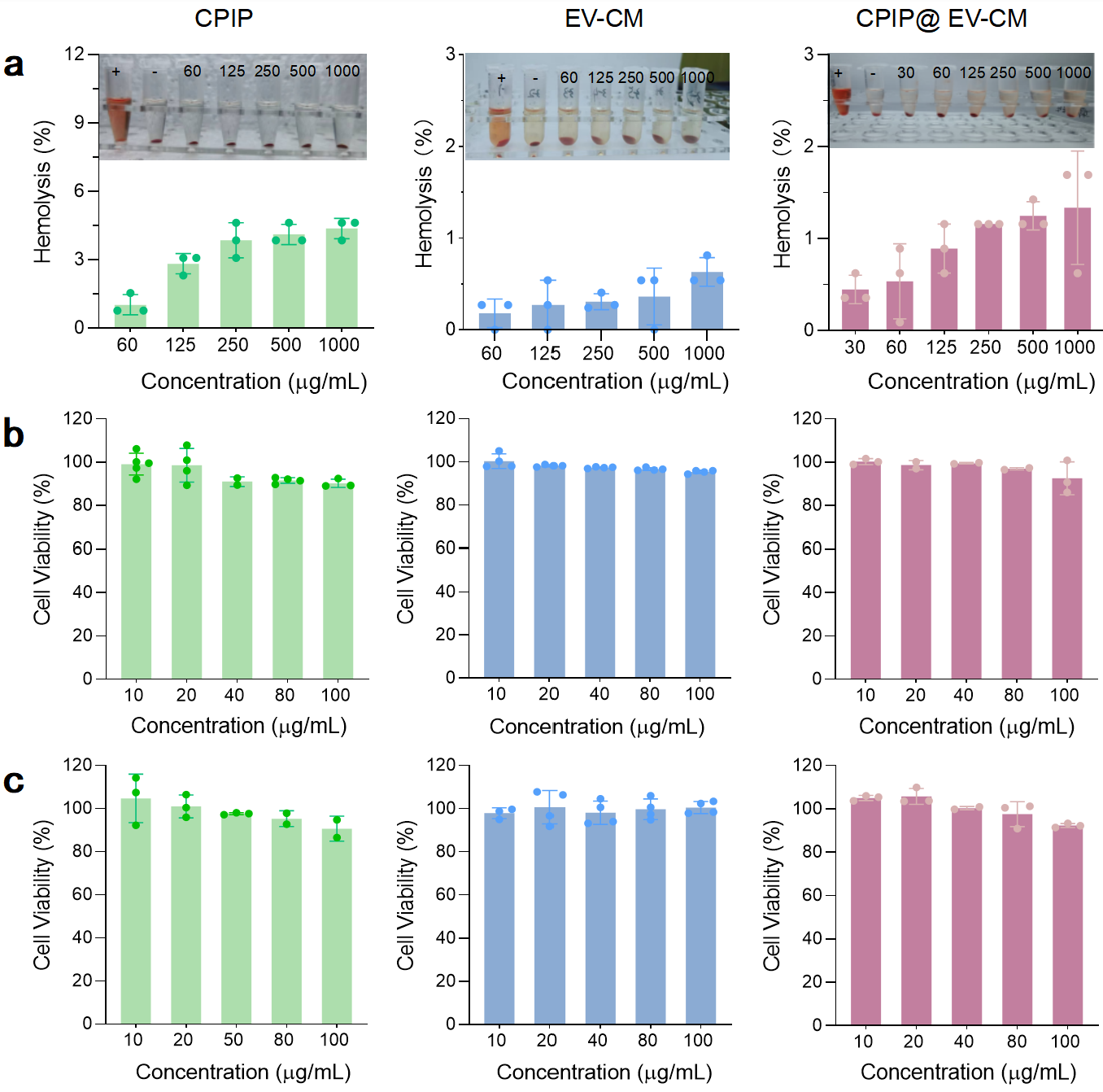


**Fig. S17 Safety testing of CPIP@EV**–**CM.**

**a** Hemolysis detection of CPIP, EV–CM, and CPIP@EV–CM at various concentrations after incubated with blood cells for 1 h. **b** HUVEC viability after incubated with CPIP, EV–CM, and CPIP@EV–CM for 24 h at different concentrations. **c** BMDC viability after incubated with CPIP, EV–CM, and CPIP@EV–CM for 24 h at different concentrations.


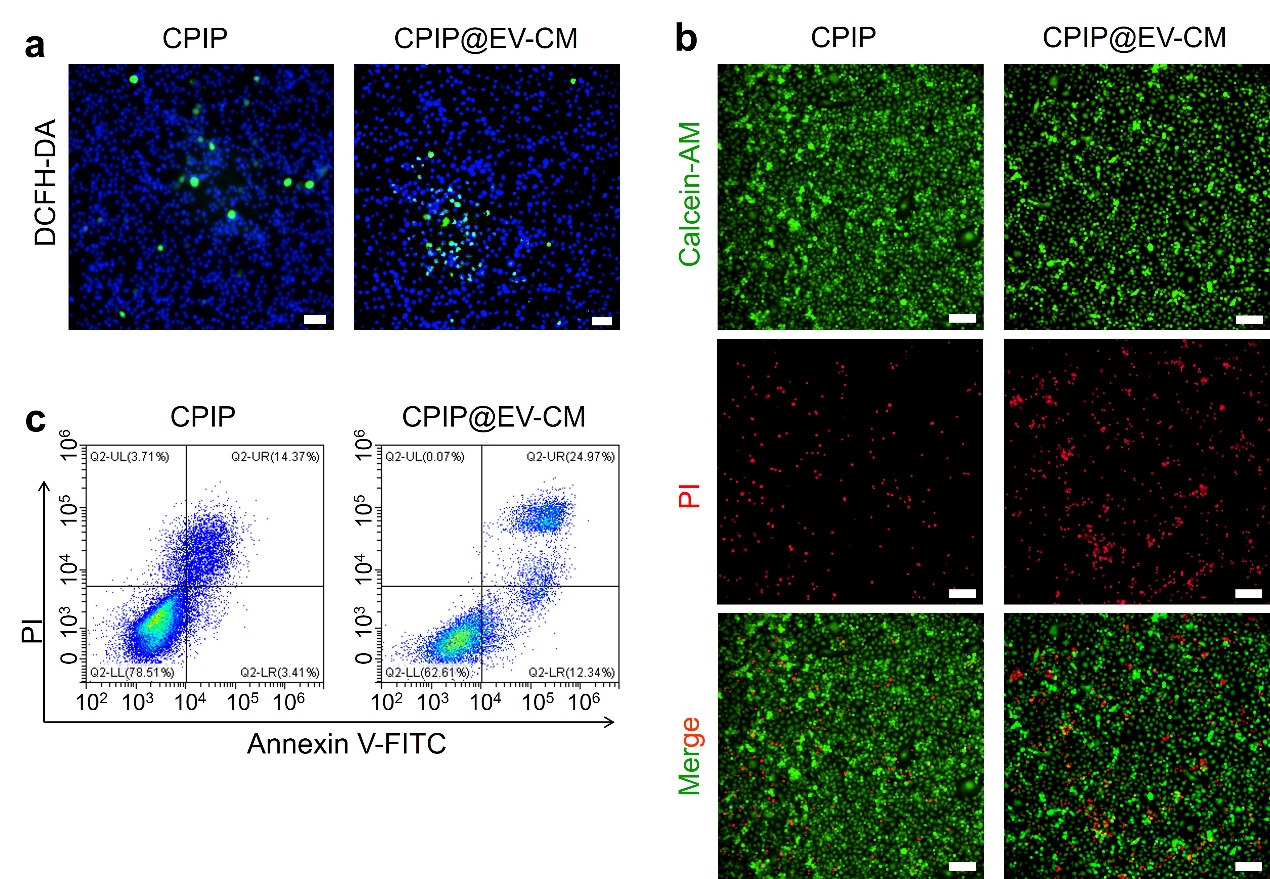


**Fig. S18 Direct tumor killing effect *in vitro* after CPIP and CPIP@EV**–**CM therapies.**

**a** Fluorescence images of DCFH-DA stained B16F10 cells after incubation with materials for 8 h (scale bar = 50 μm). **b** Fluorescence images of B16F10 cells stained with calcein-AM/PI after incubation with materials for 24 h (Scale bar = 50 μm). **c** Flow cytometric apoptosis analysis of Annexin V-FITC/PI stained B16F10 cells with different treatments for 24 h.


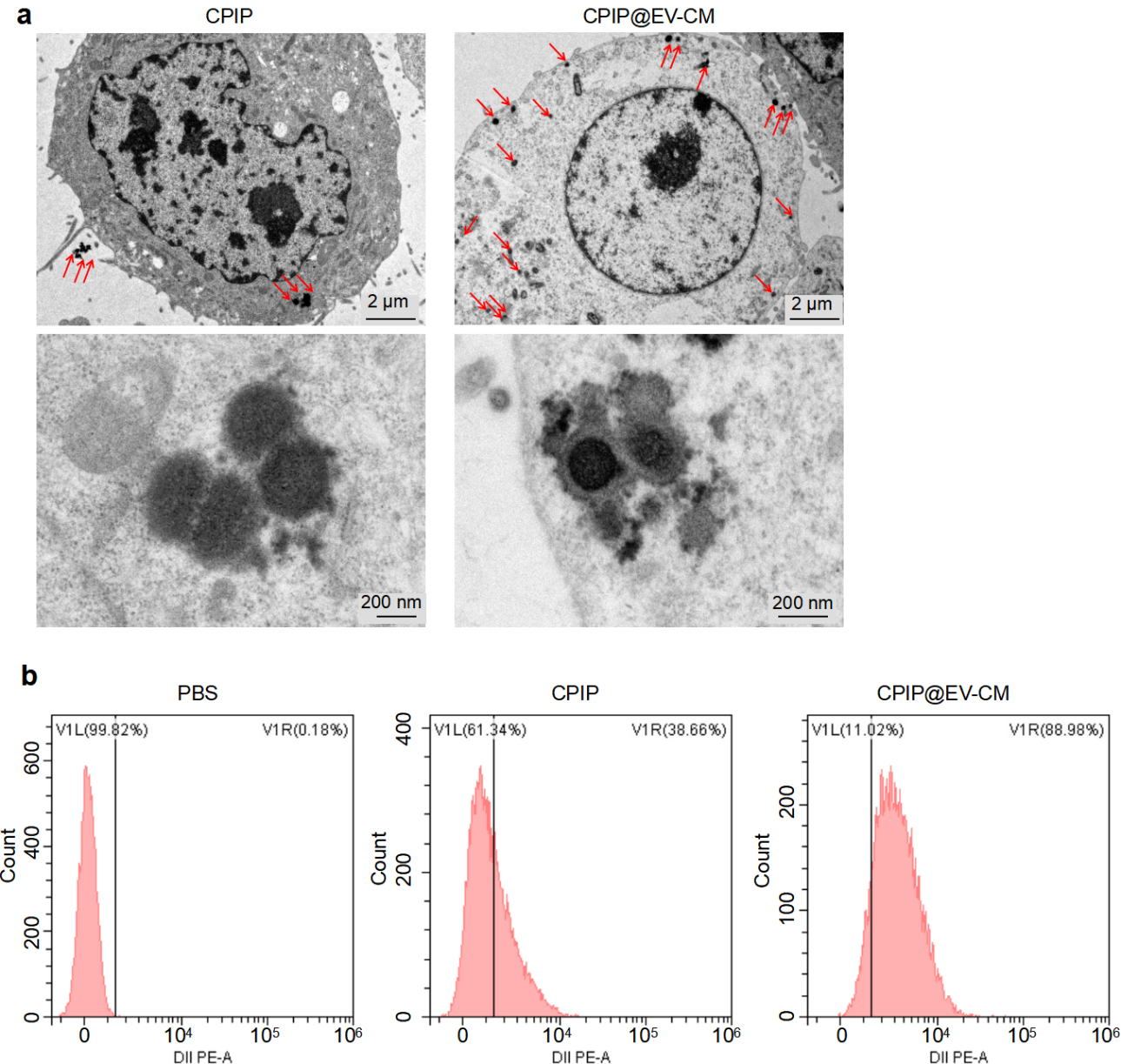


**Fig. S19 Uptake of CPIP@EV**–**CM by B16F10 cells.**

**a** Bio-TEM images of B16F10 cells treated with CPIP and CPIP@EV–CM for 6 h. **b** Flow cytometric analysis of the uptake of the nanoagents by B16F10 cells after different treatments. DiI staining nanoagents.

**
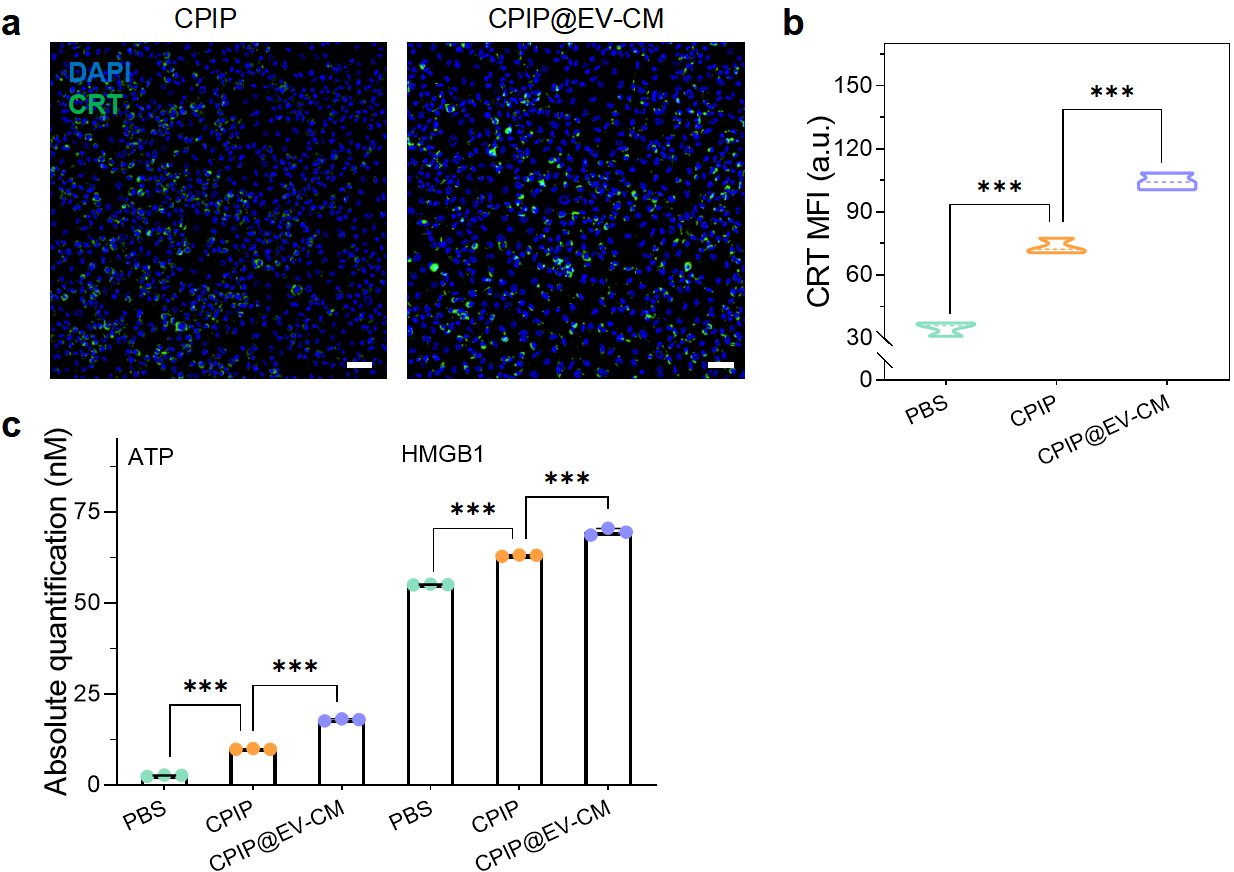
**

**Fig. S20 ICD related indicators after CPIP and CPIP@EV**–**CM therapies.**

**a** Immunofluorescence of B16F10 cells stained with CRT antibody and (**b**) the corresponding quantitative analysis. **c** Detection of ATP and HMGB1 secretion of B16F10 cells with various administration. The above tests were conducted after B16F10 cells was incubated with material for 24 h (****p* < 0.001).

**
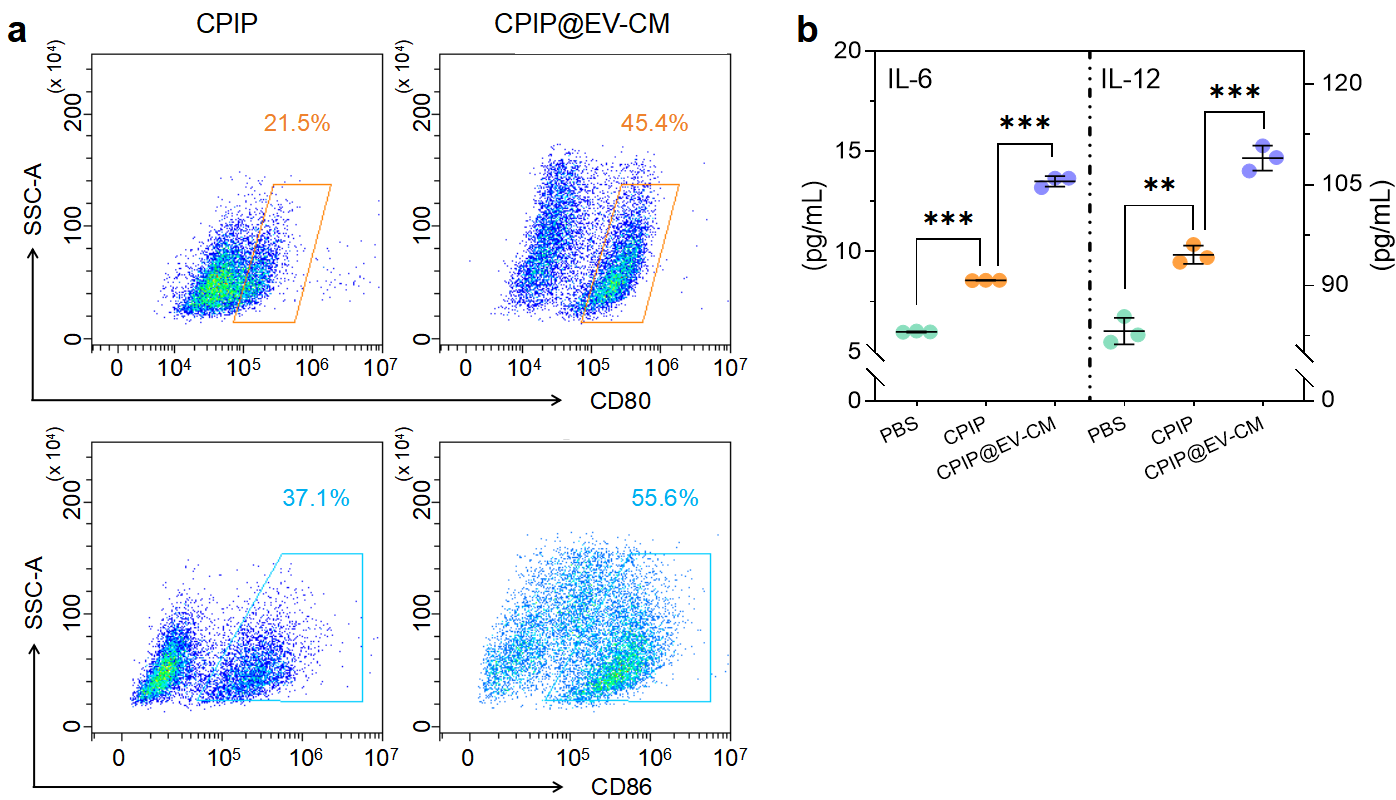
**

**Fig. S21 DC activation after *in vitro* incubation with tumor cell supernatant after different treatments.**

**a** Flow cytometric analysis of the expressed CD80 and CD86 on BMDCs. **b** Secretion of IL-6 and IL-12 in BMDCs supernatant measured by ELISA kit. The above tests were conducted after BMDCs was incubated with cell supernatant for 12 h. (****P* < 0.001).


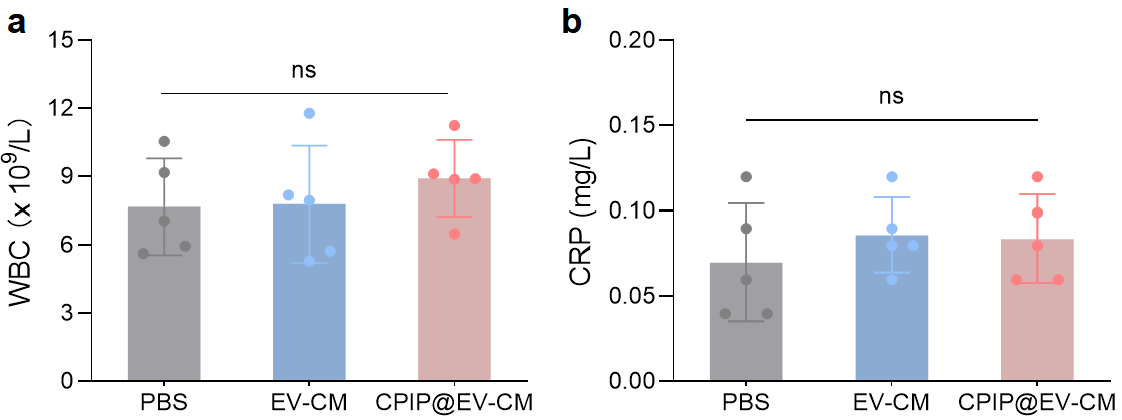


**Fig. S22 Evaluation of the risk of inflammatory response to EV preparations.**

**a** White blood cell count and serum CRP concentration (**b**) after subcutaneous injection of CPIP@EV–CM. (ns *P* > 0.05).

**
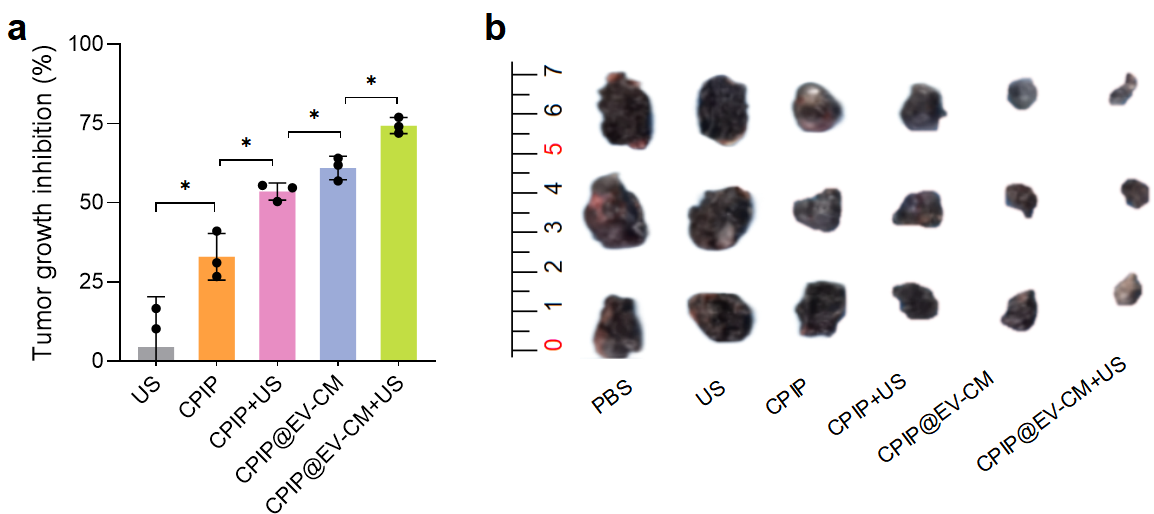
**

**Fig. S23 The therapeutic effect of orthotopic tumor.**

**a** Tumor growth inhibition on the 12th day after different treatments (**P* < 0.05). **b** Tumors explanted from the mice that received various treatments. This figure is related to Fig. 6m and Fig. S8a.

**
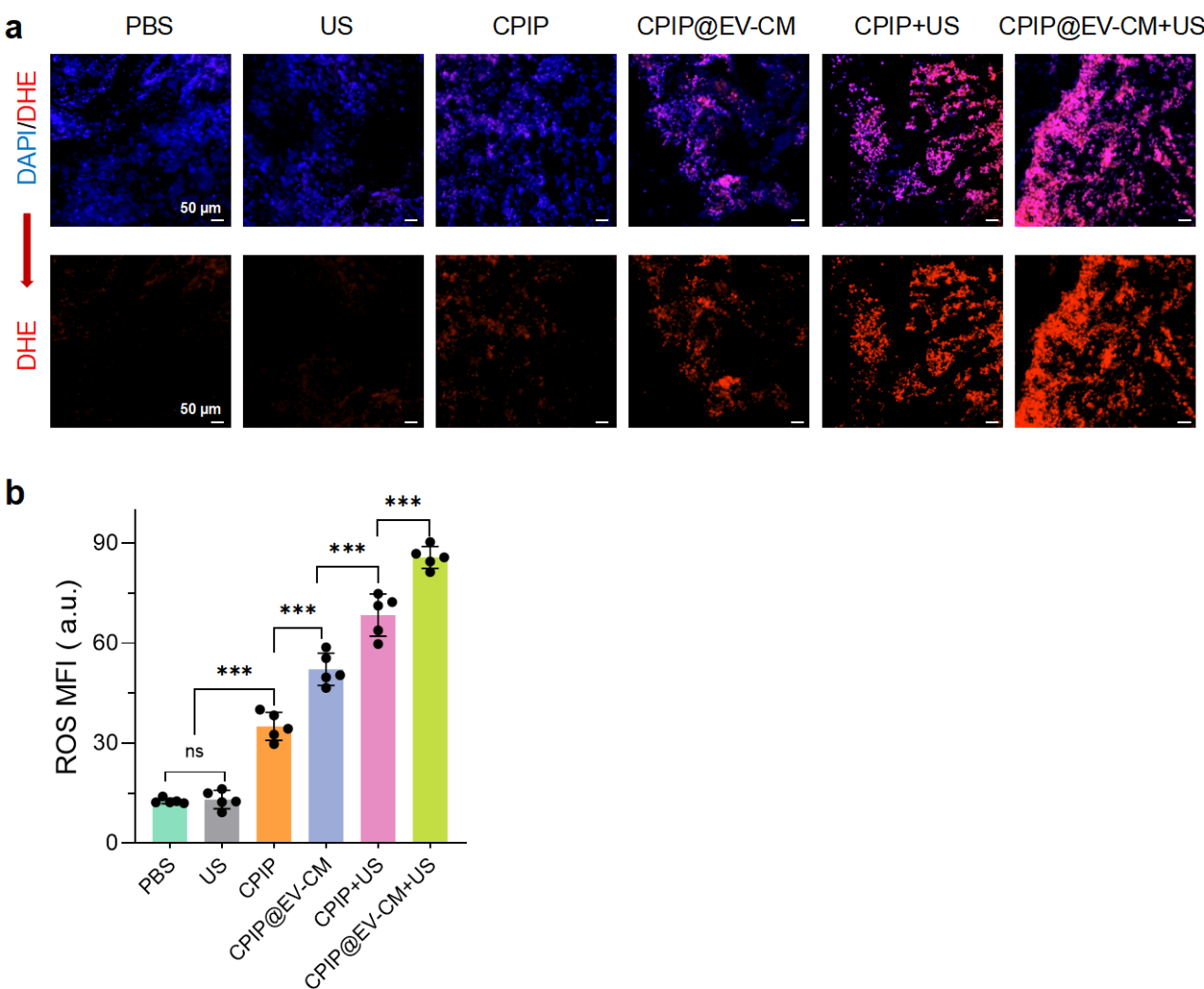
**

**Fig. S24 ROS level in tumor tissues on the 12th day of different treatment.**

**a** DHE stained tumor slices after various treatments. **b** Statistical analysis of ROS MFI after treatments (ns, nonsignificance, ****P* < 0.001).


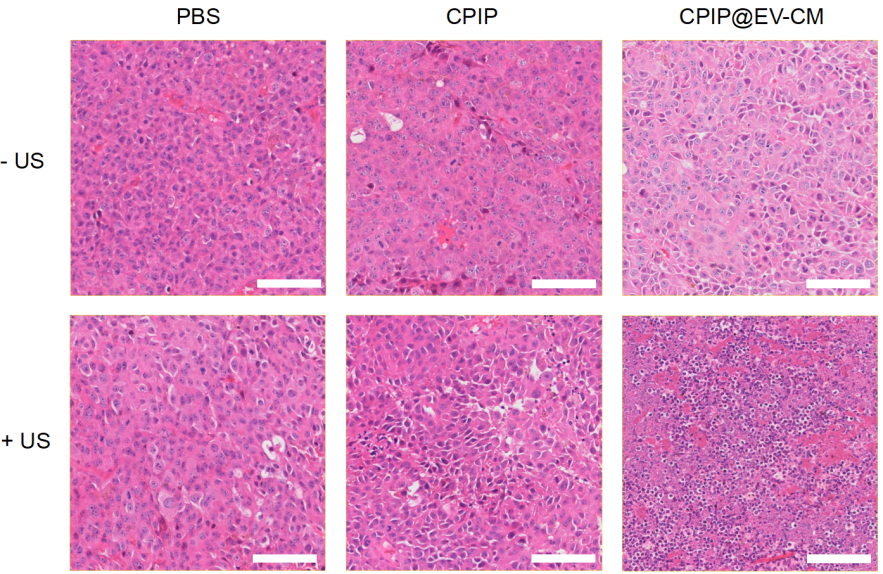


**Fig. S25 H&E staining of MM tumor slices on the 12th day after various therapies.**

CPIP@EV–CM+US group showed obviously separated, sparse and fractured tumor cells (scale bar = 100 μm). This figure is related to Fig. S8b.

**
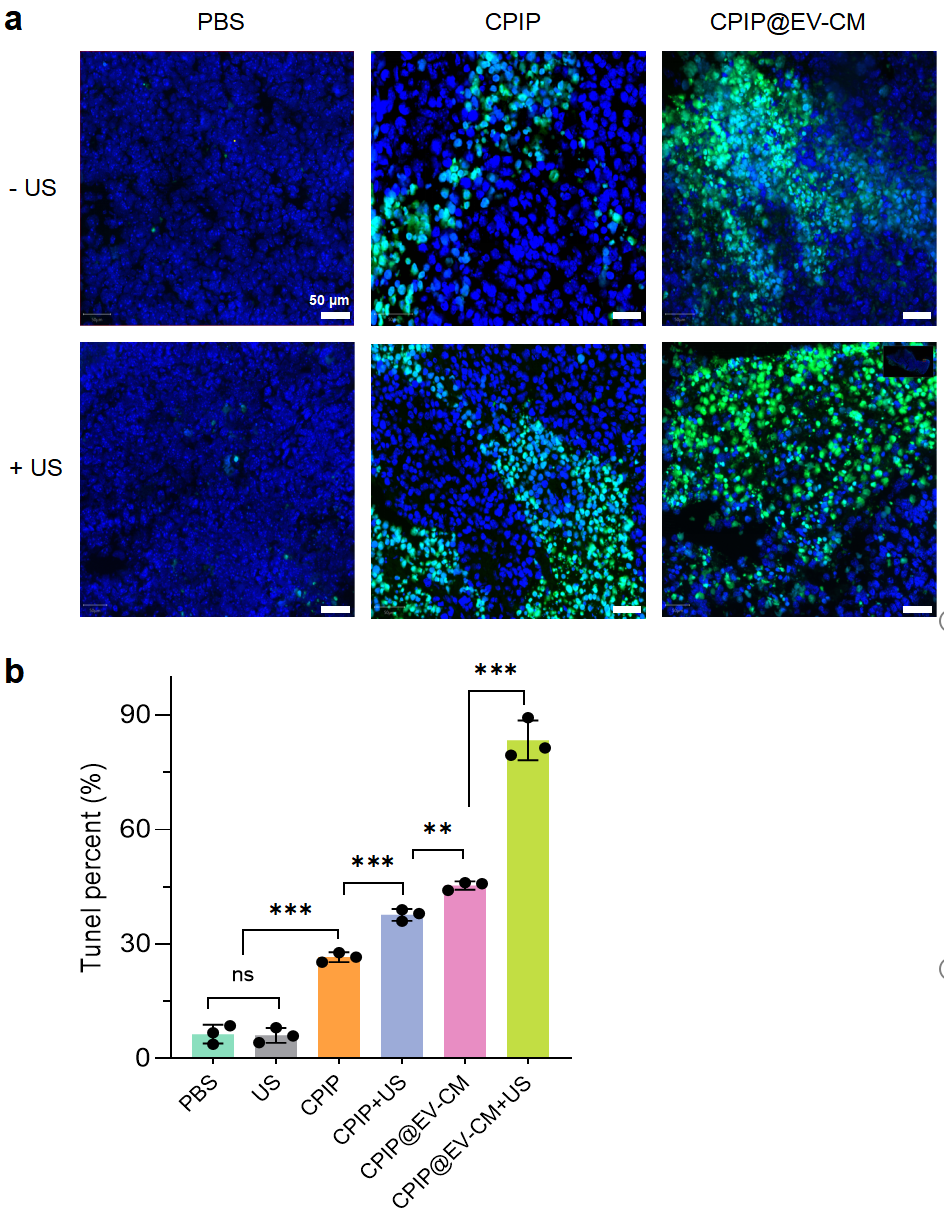
**

**Fig. S26 Detection of apoptosis rate of tumor tissue on the 12th day after different treatments.**

**a** TUNEL staining of tumor slices after various treatments (scale bar = 50 μm). and (**b**) the corresponding fluorescence quantity analysis. (ns *P* > 0.05, ***P* < 0.01, ****P* < 0.001). This figure is related to Fig. S8b.


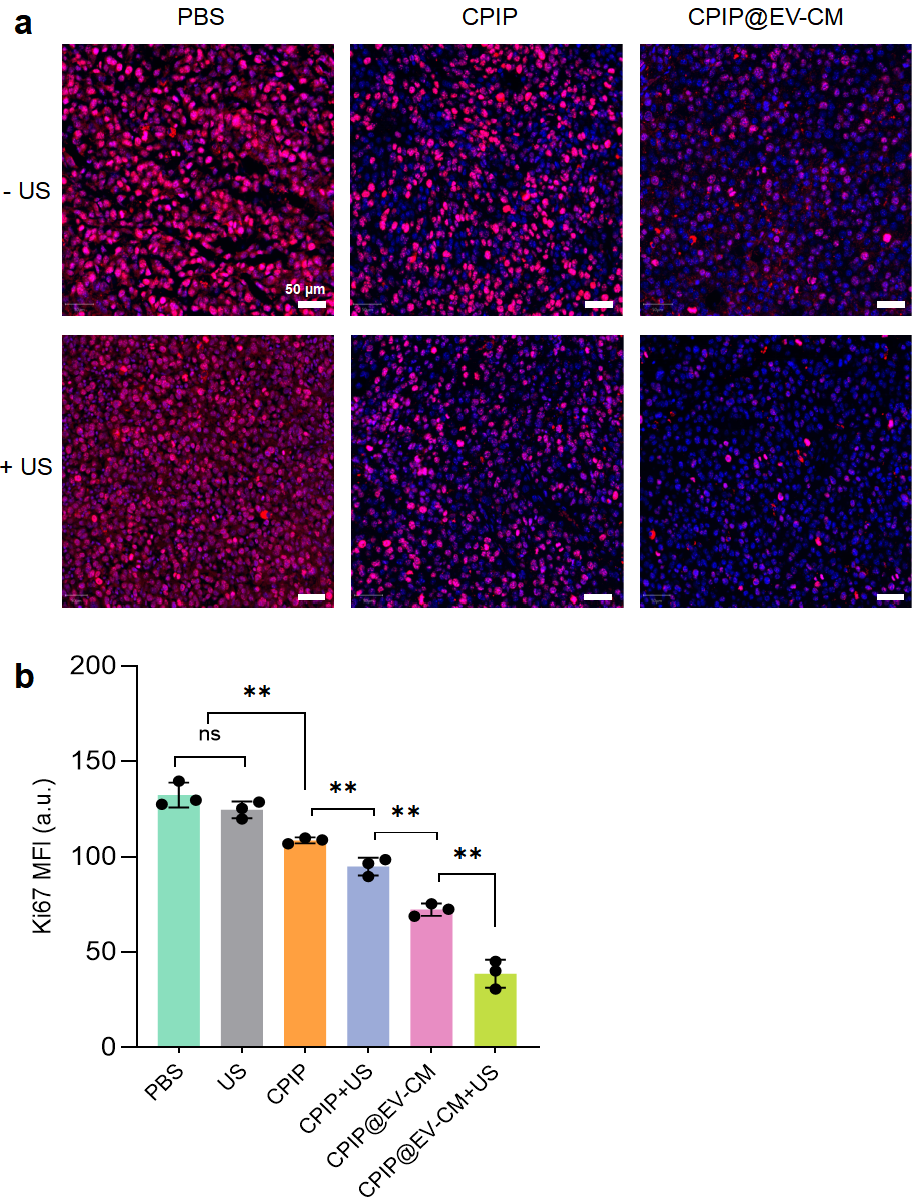


**Fig. S27 Detection of tumor proliferation dynamics on the 12th day after different treatments.**

**a** Ki67 staining of tumor slices after various treatments (scale bar = 50 μm) and (**b**) the corresponding fluorescence quantity analysis. (ns *P* > 0.05, ***P* < 0.01, ****P* < 0.001).


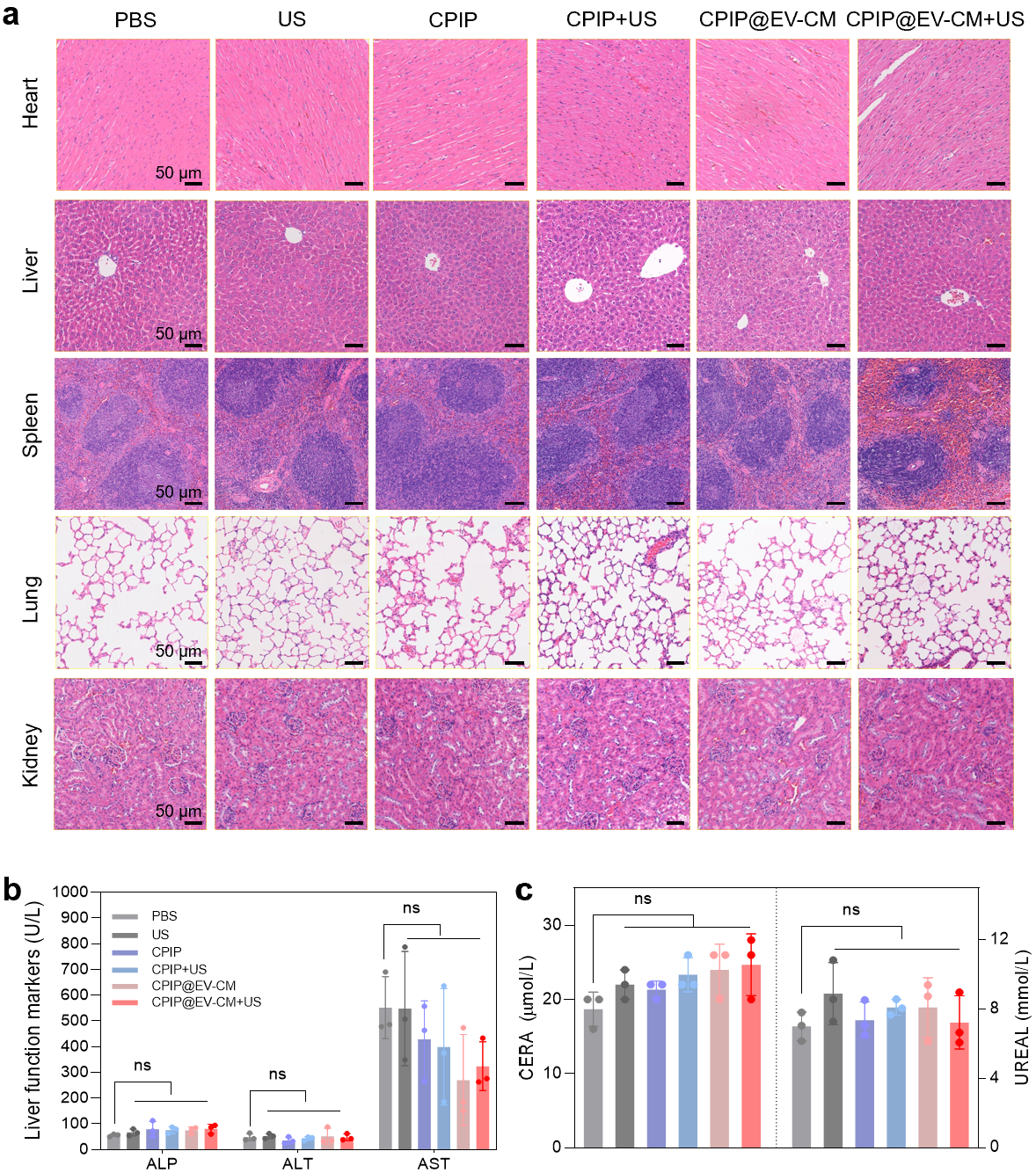


**Fig. S28 Safety testing of CPIP@EV**–**CM *in vivo*.**

**a** H&E staining of the mice's major organs (heart, liver, spleen, lung, kidney) to examine the histological changes after various treatments. **b** Blood biochemistry assays of liver function markers: ALP, ALT, AST. **c** Blood biochemistry assays of Kidney function markers: CERA, and UREAL All samples used for pathological examination and blood biochemistry assays were obtained on the 12th day after treatment intervention. (ns *P* > 0.05).


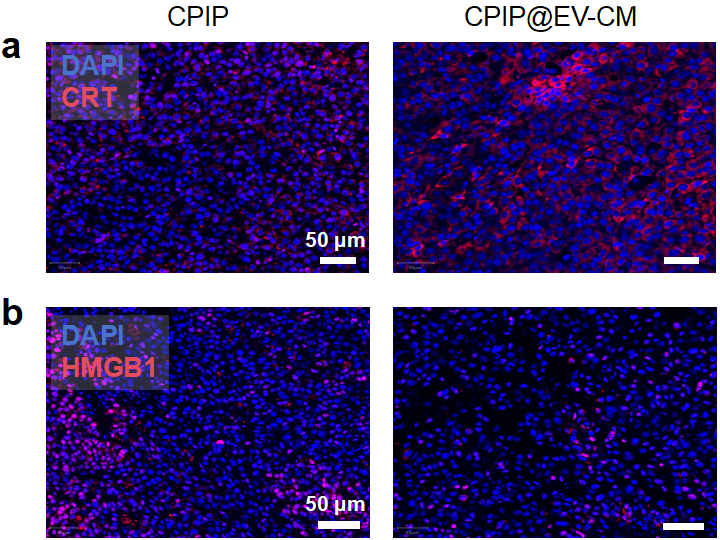


**Fig. S29 ICD related indicators of tumor tissue on the 12th day after CPIP and CPIP@EV-CM therapies.**

**a** CRT immunofluorescence staining of tumor tissue sections. **b** HMGB1 stained tumor slices after various treatments (scale bar = 50 μm).


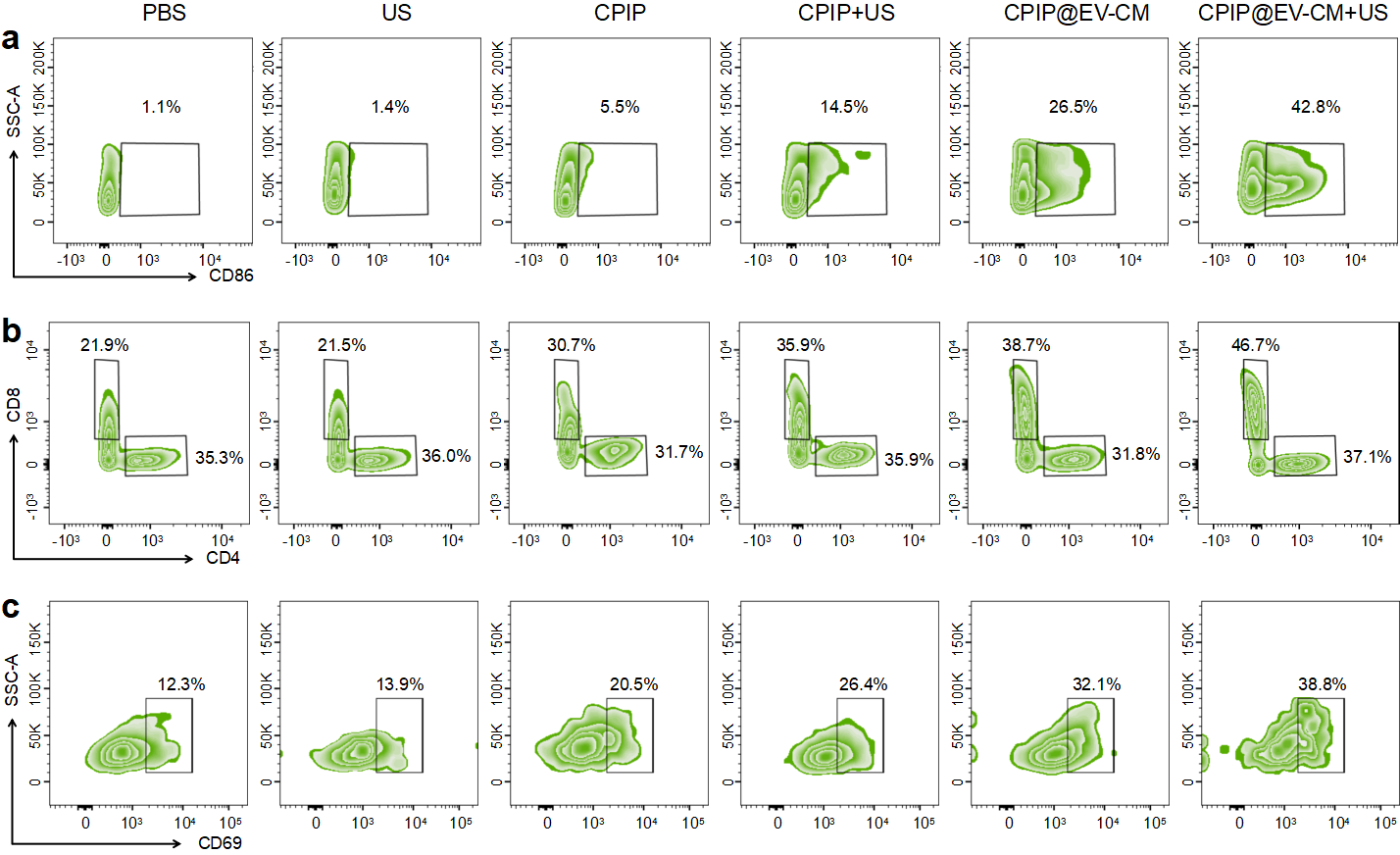


**Fig. S30** **Flow cytometric analyzed the immune activation effect of CPIP@EV**–**CM in tumors on the 12th day after treatment intervention.**

**a** Representative flow cytometry plots of mature DCs (gated on CD11c^+^ DCs). **b** Representative flow cytometry plots of T cells classification in different groups (gated on CD3^+^ T cells). **c** Representative flow cytometry plots of CD8^+^CD69^+^ T cells (gated on CD3^+^CD8^+^ T cells).

**
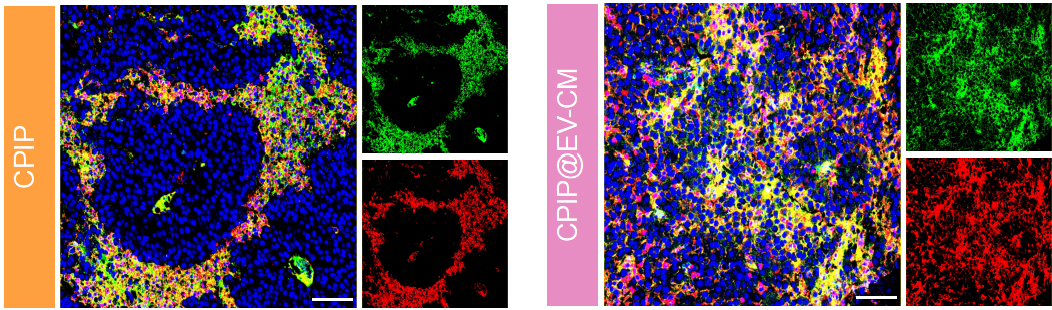
**

**Fig. S31** **Representative polychromatic immunofluorescent staining images of tumors on the 12th day after treatments.**

DAPI (blue), CD3^+^ (green), CD8^+^ (red).

**
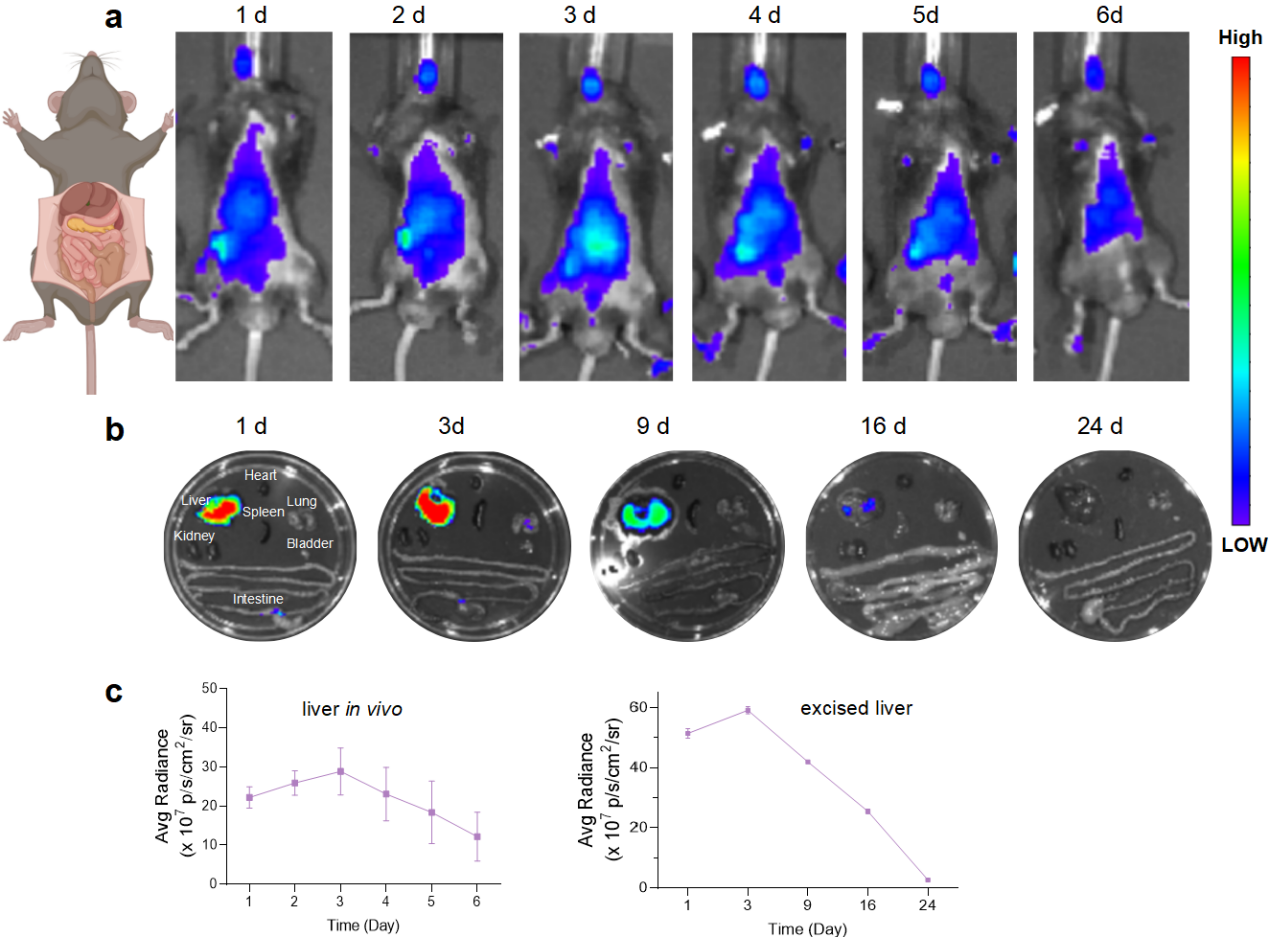
**

**Fig. S32** ***In vivo* metabolism after intratumoral injection of CPIP@EV**–**CM.**

**a** Fluorescence imaging of CPIP@EV–CM distribution in subcutaneous tumor model of B16F10. (n = 4 independent biological replicates). **b** Fluorescence imaging of the excised organs. **c** Relative quantitative analysis of liver signal intensity changes over time.


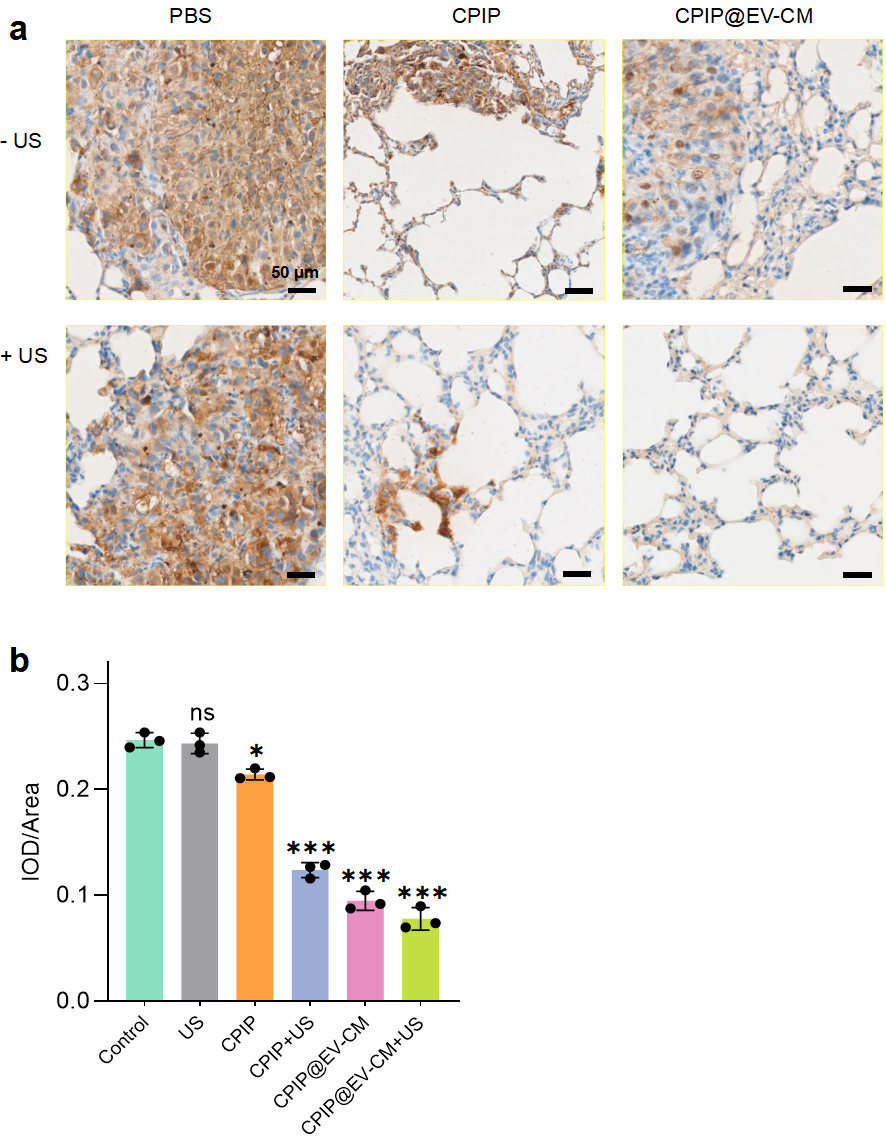


**Fig. S33** **The expression level of S100B in lung tissue on the 12th day after treatment intervention.**

**a** Representative immunohistochemical staining (scale bar = 50 μm) and (**b**) their quantity analysis (ns *P* > 0.05, **P* < 0.05, ****P* < 0.001).


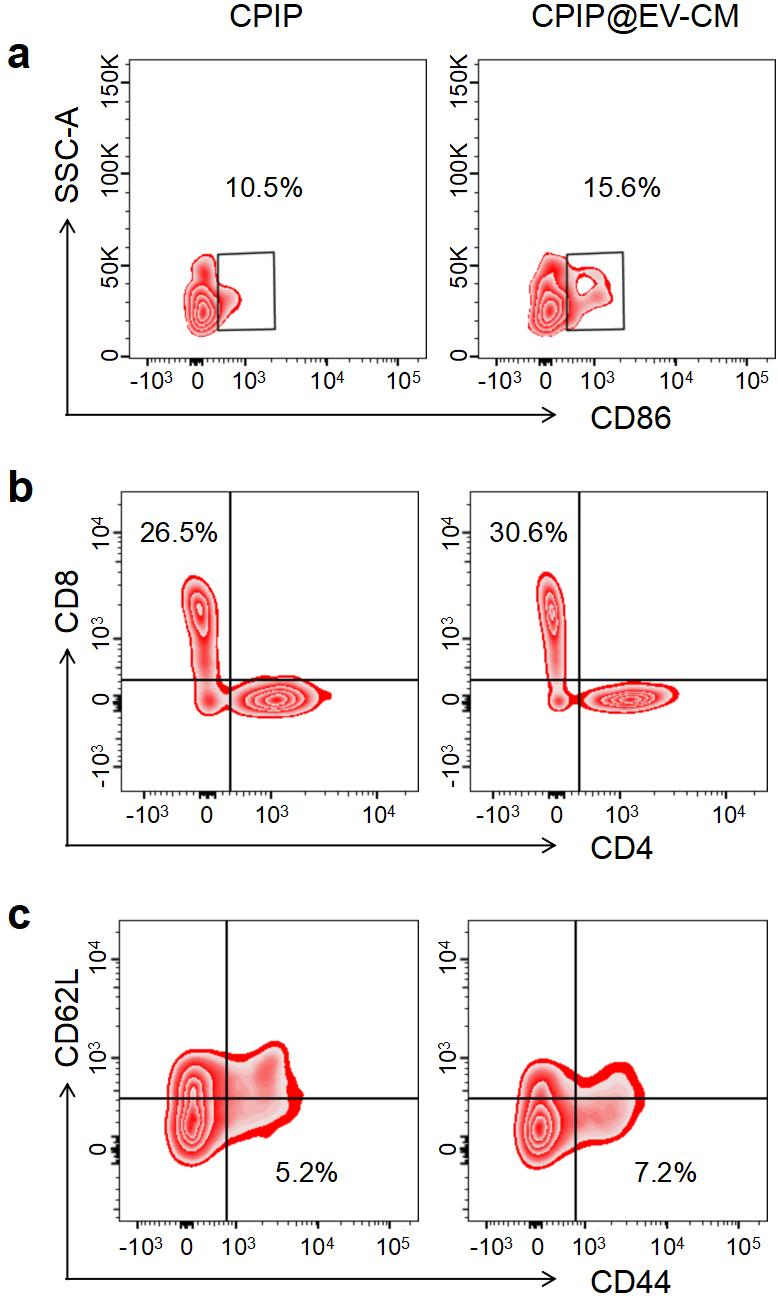


**Fig. S34 Flow cytometric analyzed the immune activation effect in lymph nodes in metastatic mouse model on the 12th day after treatment intervention.**

**a** Representative flow cytometry plots of CD86^+^ DC cells (gated on CD11c DC cells). **b** Representative flow cytometry plots of CD3^+^CD8^+^ T cells (gated on CD3^+^ T cells). **c** Representative flow cytometry plots of CD44^+^CD62L^-^ T cells (gated on CD3^+^CD8^+^ T cells).


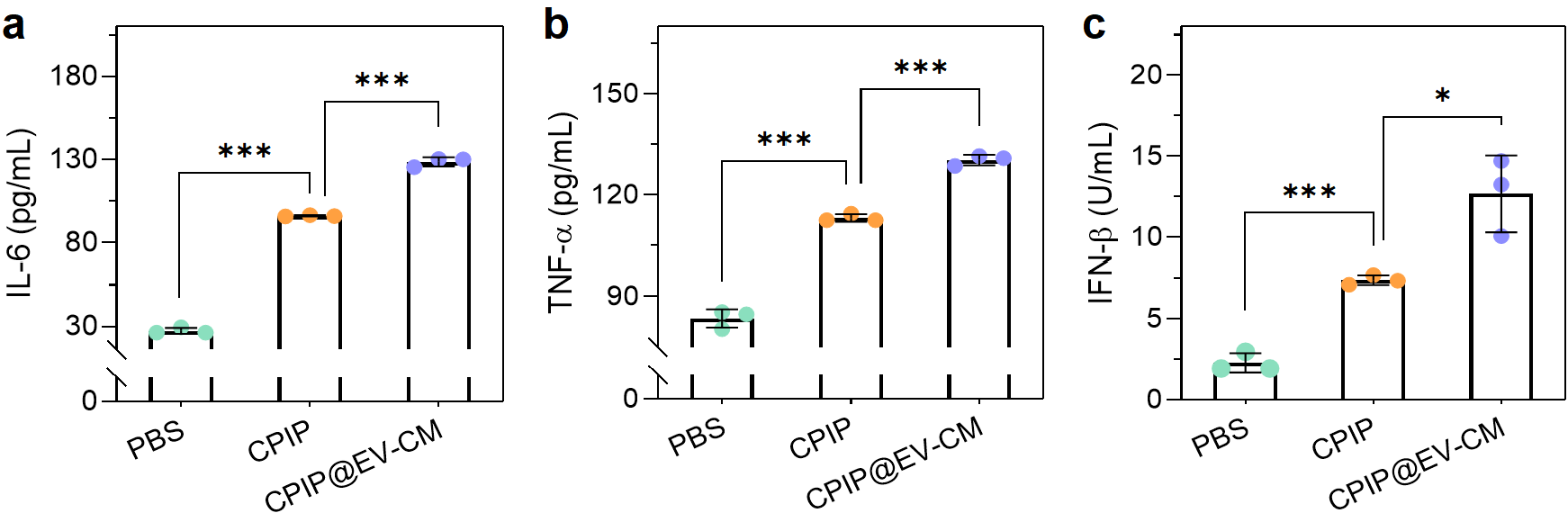


**Fig. S35** **The concentrations of IL-6, TNF-α, and IFN-β tested by ELISA.**

Concentrations of IL-6, TNF-α, and IFN-β in blood serum on the 12th day after different treatments. (**P* < 0.05, ****P* < 0. 001).

**
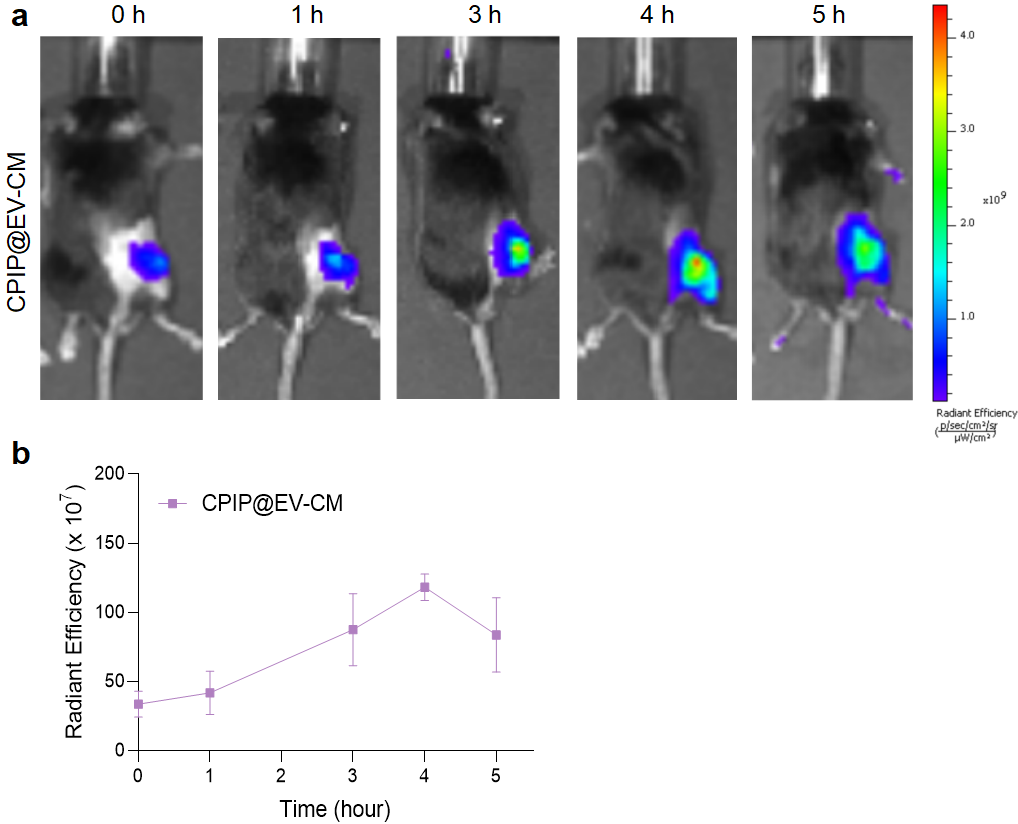
Fig. S36** **The metabolism of CPIP@EV**–**CM at the tumor site after intratumoral injection.**

**a** *In vivo* fluorescence imaging of tumor-bearing mice after intratumor injection. **b** Attenuation curve of fluorescence signal within the tumor. (n = 4 per group. data are presented as mean ± SD)

**References**

1. Du, F.*, et al.* Pd-Single-Atom Coordinated Biocatalysts for Chem-/Sono-/Photo-Trimodal Tumor Therapies. *Adv. Mater.* **33**, 2101095 (2021).

2. Duan, S.*, et al.* Oncolytic Virus-Driven Biotherapies from Bench to Bedside. *Small* **19**, e2206948 (2023).

3. Corsi, M.M., Sandberg, J.K., Wasserman, K., Maes, H.H. & Kiessling, R. Generation and function of bone marrow-derived dendritic cells from CD4/CD8 −/− double-knockout mice. *Immunol. Lett.* **67**, 243-249 (1999).
